# Genetic structure and forensic characteristics of Tibeto-Burman-speaking Ü-Tsang and Kham Tibetan Highlanders revealed by 27 Y-chromosomal STRs

**DOI:** 10.1038/s41598-019-44230-2

**Published:** 2019-05-23

**Authors:** Guanglin He, Zheng Wang, Yongdong Su, Xing Zou, Mengge Wang, Xu Chen, Bo Gao, Jing Liu, Shouyu Wang, Yiping Hou

**Affiliations:** 1 Institute of Forensic Medicine, West China School of Basic Medical Sciences & Forensic Medicine, Sichuan University, Chengdu, Sichuan 610041 China; 2 Forensic Identification Center, Public Security Bureau of Tibet Tibetan Autonomous Region, Lhasa, Tibet Tibetan Autonomous Region 850000 China; 3 Department of Clinical Laboratory, the First People’s Hospital of Liangshan Yi Autonomous Prefecture, Xichang, Sichuan 615000 China; 4 Yili Public Security Bureau, Yili, Xinjiang Uygur Autonomous Region 418000 China

**Keywords:** Evolutionary genetics, Genetic variation

## Abstract

Culturally diverse Tibetans (Ü-Tsang, Kham and Ando) harboring a unique molecular mechanism that allows them to successfully adapt to hypoxic environments in the Qinghai-Tibet Plateau have been a subject of great interest in medical genetics, linguistics, archeology and forensic science. However, forensic characteristics and genetic variations of the Y-chromosomal 27-marker haplotype included in the Yfiler Plus system in the Ü-Tsang and Kham Tibeto-Burman-speaking Tibetans remain unexplored. Thus, we genotyped 27 Y-STRs in 230 Shigatse Ü-Tsang Tibetans (SUT) and 172 Chamdo Kham Tibetans (CKT) to investigate the forensic characterization and genetic affinity of Chinese Tibetan Highlanders. The haplotype diversities were 0.999962028 in SUT and 0.999796002 in CKT. Forensic diversity measures indicated that this 27-Y-STR amplification system is appropriate for routine forensic applications, such as identifying and separating unrelated males in deficiency paternity cases, male disaster victims and missing person identification and determining male components in sexual assault cases. Moreover, the genetic relationships among 63 worldwide populations (16,282 individuals), 16 Asian populations, and 21 Chinese populations were analyzed and reconstructed using principal component analysis, multidimensional scaling plots and a phylogenetic tree. Considerable genetic differences were observed between Tibetan populations and other geographically/ethnically diverse populations (Han Chinese). Our studied SUT and CKT have a genetically closer relationship with Gansu Ando Tibetans than with other Asians. In total, our analyses indicated that subpopulation structures exist among Asian and Chinese populations, and population-specific reference databases should be established for forensic applications.

## Introduction

The Qinghai-Tibet Plateau, also referred to as the Himalayan Plateau, stretches approximately 1,000 kilometers (km) from the Kunlun Mountains and Qilian Mountains in the north to the inner Himalayan Range in the south and 2,500 km from northwestern Yunnan and western Sichuan in the east to the rugged Karakoram Range of northern Kashmir in the west. This elevated plateau with an average elevation exceeding 4,500 meters harbors the world’s highest summits (Mount Everest) and is called the Roof of the World. Tibetans, with a population of over 7.5 million, settled in the Qinghai-Tibet Plateau and other parts of China. There are also significant numbers of Tibetans (approximately 1.5 million) residing in India, Pakistan, Bhutan, Nepal and other countries. Ancestry history and genetic origin as well as the processes of Tibetan adaptation in extreme environments (high altitude, low temperature, hypobaric hypoxia and high ultraviolet radiation) have attracted the attention of archeologists, anthropologists, and geneticists^1–3^. Archeological evidence suggested that the initial stage occupation of the Tibetan Plateau occurred 40–25 thousand years ago (kya)^4^. Y-chromosome evidence based on the haplogroup distribution of D-M174 found that the earliest modern human settlement and migration in East Asia occurred approximately 60 kya with multiple origins of Tibetans and complex admixture occurring during the subsequent northward migration^5^. Maternal fine-scale mitochondrial genome variations revealed that Tibetan-specific mtDNA lineages entered the Tibetan Plateau in both the Late Paleolithic and mid-Holocene (M16)^6^. Recently, new geological, paleoclimatological and archeological evidence suggested that the earliest occupations of the Tibetan Plateau could date back as early as 30 kya^2^. Recently, novel genetic findings via Y-chromosomal, mitochondrial and genome-wide autosomal markers demonstrated the Upper Paleolithic colonization and Neolithic expansion of anatomically modern humans in Tibet^7^, as well as the coexistence of Paleolithic and Neolithic ancestries in modern Tibetans^8^. Early whole-genome association studies have successfully identified that the genetic variants in the high-altitude adaptation genes (EPAS1 and EGLN1) are correlated significantly with hemoglobin concentration^9–14^. Additional genetic research indicated that at least three evolutionary mechanisms, including admixture with surrounding Highlanders, interbreeding with archaic Denisovans, and natural selection, have driven Tibetans to adapt to the extreme environments^15–18^.

In addition, there are several subgroups with different cultural backgrounds in the Tibetan population separated by the Alpine and Gorge Regions, including Kham Tibetans, Ü-Tsang Tibetans and Ando Tibetans. Kham Tibetans settled in the rugged terrain located at the boundary of the eastern region in the Tibet Autonomous Region, the western region of Sichuan Province and part of the southern region of Qinghai and Gansu Provinces as well as smaller portions of the northern region of Yunnan Province. Linguists and anthropologists regard this region as the Tibetan-Yi Corridor or Ethnic Corridor of Southwest China due to the complex landscapes of culturally and linguistically distinct ethnic groups. Most of the people residing in the Tibetan-Yi Corridor speak the Qiangic language as their mother language; however, Kham Tibetans speak the Tibetic language. Ü-Tsang Tibetans are also called central Tibetans, Dbus, or Ü Tibetans and mainly reside in the central and western region of the Tibet Autonomous Region. Ü-Tsang Tibetan use the standard conservative Tibetan language. Ando Tibetans mainly live in Qinghai Province and some regions of the Sichuan and Gansu Provinces. Their language is Andolese. Zhang *et al*. recently identified the differentiated population genetic structure among the aforementioned geographically different Tibetans^3^. Thus, a clear elucidation and understanding of the genetic polymorphisms and forensic characteristics of forensic genetic markers (maternal mitochondrial DNA, paternal Y-chromosomal and autosomal STRs) are necessary and important.

Y-chromosome variations situated within the nonrecombining region, which are paternally inherited and haploid, have received increased attention as classical genetic markers in evolutionary and forensic genetic studies, such as tracing and reconstructing the paternal population history and evaluating population structure and relationships^19,20^. Approximately 4,500 Y-chromosome short tandem repeats (Y-STRs) have been reported and subsequently used in reconstructing human male population evolutionary history and other multidisciplinary research^21^. In the forensic field, Y-STRs can be used in paternal relationship identification in deficiency paternity cases, male disaster victims and missing person identification and can help determine the male components in sexual assault cases and family research in forensics^22^. In the past decade, many commercial amplification systems that include 17 to 24 Y-STR loci, for instance, Yfiler (17 Y-STRs), PowerPlex Y23 (23 Y-STRs), AGCU Y24 and GFS Y24 (24 Y-STRs), have been developed and used in forensics and population genetics^23–26^. Recently, a next-generation Y-STR genotyping system comprising 27 Y-STR loci, including all 17 loci (DYS456, DYS438, DYS458, DYS635, DYS439, DYS448, DYS389I, DYS19, DYS391, DYS392, DYS437, DYS389II, DYS390, DYS393, DYS385a/b and Y-GATA H4) included in the Yfiler kit, seven rapidly mutating Y-STR loci (DYF387S1a/b, DYS449, DYS518, DYS570, DYS576 and DYS627)^27^, and three highly polymorphic Y-STR loci (DYS460, DYS481 and DYS533)^28^ was developed for forensic Y-STR database establishment and forensic routine applications. Current population genetic studies have so far focused on a limited number of ethnically, geographically and linguistically restricted populations or targeted autosomal and X-chromosomal markers^29–34^.

However, existing genetic data from the Y-chromosome of Tibetan Highlanders (only Gansu and Qinghai Ando Tibetans) are insufficient to investigate forensic features and characterize the complex genetic relationships between and within Tibetans and other East Asians^35^. To gain insights into the genetic diversity and forensic characteristics of forensic Y-chromosomal genetic markers, we genotyped and analyzed 27 Y-STRs in 230 Shigatse Ü-Tsang Tibetans (SUT) and 172 Chamdo Kham Tibetans (CKT). Subsequently, we performed comprehensive population comparisons corresponding to cultural, geographical or linguistic groups to dissect the genetic similarities and differences between our studied Tibetan populations and Chinese reference populations or other adjacent Tibetan-Burman speaking populations^20,35–42^.

## Results and Discussion

The Y-chromosomal haplotype reference database (YHRD) was developed 16 years ago and is a robust and reliable resource to revisit the worldwide or regional male genetic landscape, dissect Y-specific population differentiation and evaluate the forensic characteristics. Currently, this database includes 77,805 Yfiler haplotypes (17-Y-STRs), 12,430 PowerPlex Y23 haplotypes (23-Y-STRs) and 26,933 Yfiler Plus haplotypes (27-Y-STRs) from China. China is the world’s most populous country, consisting of 56 ethnicities from more than 30 administrative divisions. However, no Yfiler Plus haplotype data were available for CKT and SUT residing in the Tibet Tibetan autonomous region. In this study, we first examined the patterns of Y-chromosome variations in these two culturally different Tibetan populations and investigated the genetic heterogeneity and homogeneity between Tibetan populations and other Asian, nationwide or Himalayan-related reference populations^20,35–41^.

### Genetic polymorphisms and forensic characteristics of 27 Y-STRs in Tibetans

**Figure 1:**
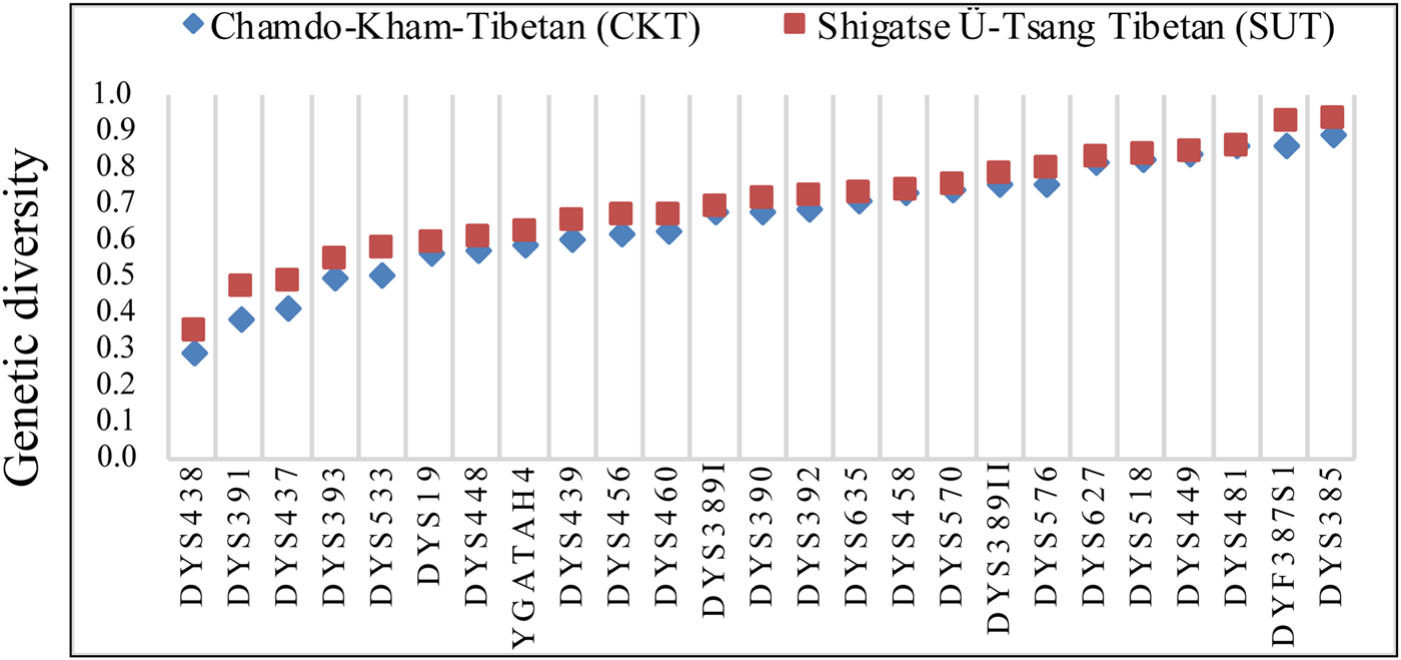
Genetic diversity (GD) of 27 Y-chromosomal STRs included in the Yfiler Plus amplification system in two studied Tibeto-Burman-speaking Ü-Tsang and Kham Tibetan Highlanders.

In total, 230 SUT and 172 CKT subjects were successfully genotyped, and the corresponding genotype data and haplotype data are presented in Supplementary Table S1. In addition, all our raw genotypes were submitted to the YHRD with accession numbers of YA004561 (CKT) and YA004562 (SUT). Three duplicate alleles at three single-copy loci (15, 17 at DYS19 locus; 18, 19 at DYS576 locus; 18, 19 at DYS570 locus) were observed. Intermediate alleles of 19.2 at the DYS627 locus in one SUT and 37.2 at the DYS518 locus in four CKT people were detected. The allele frequencies and genetic diversity of 23 single-copy Y-STR loci and two multicopy loci are presented in Fig. 1 and Supplementary Tables S2 and 3. A total of 144 alleles with corresponding frequencies ranging from 0.0058 to 0.8256 in CKT and 160 alleles with corresponding allele frequencies ranging from 0.0043 to 0.7826 in SUT were observed in the 23 single-copy loci. GD values spanned from 0.2936 (DYS438) to 0.8630 (DYS481) in CKT and from 0.3512 (DYS391) to 0.8625 (DYS518) in SUT. Twenty-eight allelic combinations with 9 different alleles at the DYF387S1a/b locus and 47 allelic combinations with 13 different alleles at the DYS385a/b locus in SUT were observed. In addition, we detected 18 allelic combinations with 7 different alleles at the DYF387S1a/b locus and 31 allelic combinations with 12 distinct alleles at the DYS385a/b locus in CKT.

Among 172 CKT genotypes, 169 different haplotypes consisting of 166 unique haplotypes and three duplicated haplotypes were detected. The match probability (MP) and HD were 0.0060 and 0.999796, respectively. A total of 229 different haplotypes were observed in 230 SUT, of which 228 were unique and one was a duplicate. The MP and HD were 0.0044 and 0.999962, respectively. To comprehensively evaluate the forensic discrimination power of different marker compositions in three culturally different Tibetan populations, we combined our newly genotyped data with previously published Yfiler Plus haplotype data that included 27 Y-STRs from the Qinghai Ando Tibetan population^35^. The forensic parameters of different marker sets, including the minimal haplotype, the extended haplotype, the PowerPlex Y haplotype, and the Yfiler and Yfiler^®^ Plus PCR Amplification systems in CKT, SUT and previously reported Qinghai Ando Tibetans^35^, are presented in Table 1. The results indicated that the forensic discrimination power increases when the number of analyzed markers increases, especially the six included rapidly mutating Y-STRs^43^. This Y-STR genotyping system is discriminating and polymorphic in the different Tibetan populations with different cultural backgrounds and is suitable as a powerful tool for identifying paternal lineages, linking suspects and crime samples and conducting forensic family research in Tibetan populations.

**Table 1:** The forensic parameters estimated for different combinations of Y-STRs in three different Tibetan populations

No. of observed haplotypes	CKT					SUT					Qinghai Ando Tibetan				
	MHT	EXT	PPY12	Yfiler	Yfiler Plus	MHT	EXT	PPY12	Yfiler	Yfiler Plus	MHT	EXT	PPY12	Yfiler	Yfiler Plus
1(unique)	76	86	86	117	166	147	160	169	210	228	426	430	432	434	434
2	10	9	11	21	3	17	17	17	10	1	38	36	35	34	34
3	6	8	8	3		3	5	3			3	3	3	3	3
4	4	4	4	1		5		3							
5	1					1	3								
6		1	2			1	1	1							
9						1									
10	1	1													
11	1														
12		1	1												
16	1														
Sample Size	172	172	172	172	172	230	230	230	230	230	511	511	511	511	511
No. of haplotypes	100	110	112	142	169	175	189	193	220	229	467	469	470	471	471
Fraction of unique haplotypes	0.4419	0.5000	0.5000	0.6802	0.9651	0.6391	0.6957	0.7348	0.9130	0.9913	0.8337	0.8415	0.8454	0.8493	0.8493
Match probability	0.0249	0.0182	0.0163	0.0082	0.0060	0.0088	0.0073	0.0066	0.0047	0.0044	0.0023	0.0023	0.0023	0.0023	0.0023
Haplotype diversity	0.9808	0.9876	0.9895	0.9976	0.9998	0.9956	0.9971	0.9978	0.9996	>0.9999	0.9996	0.9997	0.9997	0.9997	0.9997

MHT: minimal haplotype; EHT: the extended haplotype; PPY12: PowerPlex Y haplotype; Yfiler: Yfiler PCR Amplification Kit; Yfiler Plus: Yfiler® Plus PCR Amplification Kit. SUT: Shigatse Ü-Tsang Tibetans (SUT), CKT: Chamdo Kham Tibetans.

### Worldwide population genetic relationship inferred from the raw genotype data

**Figure 2:**
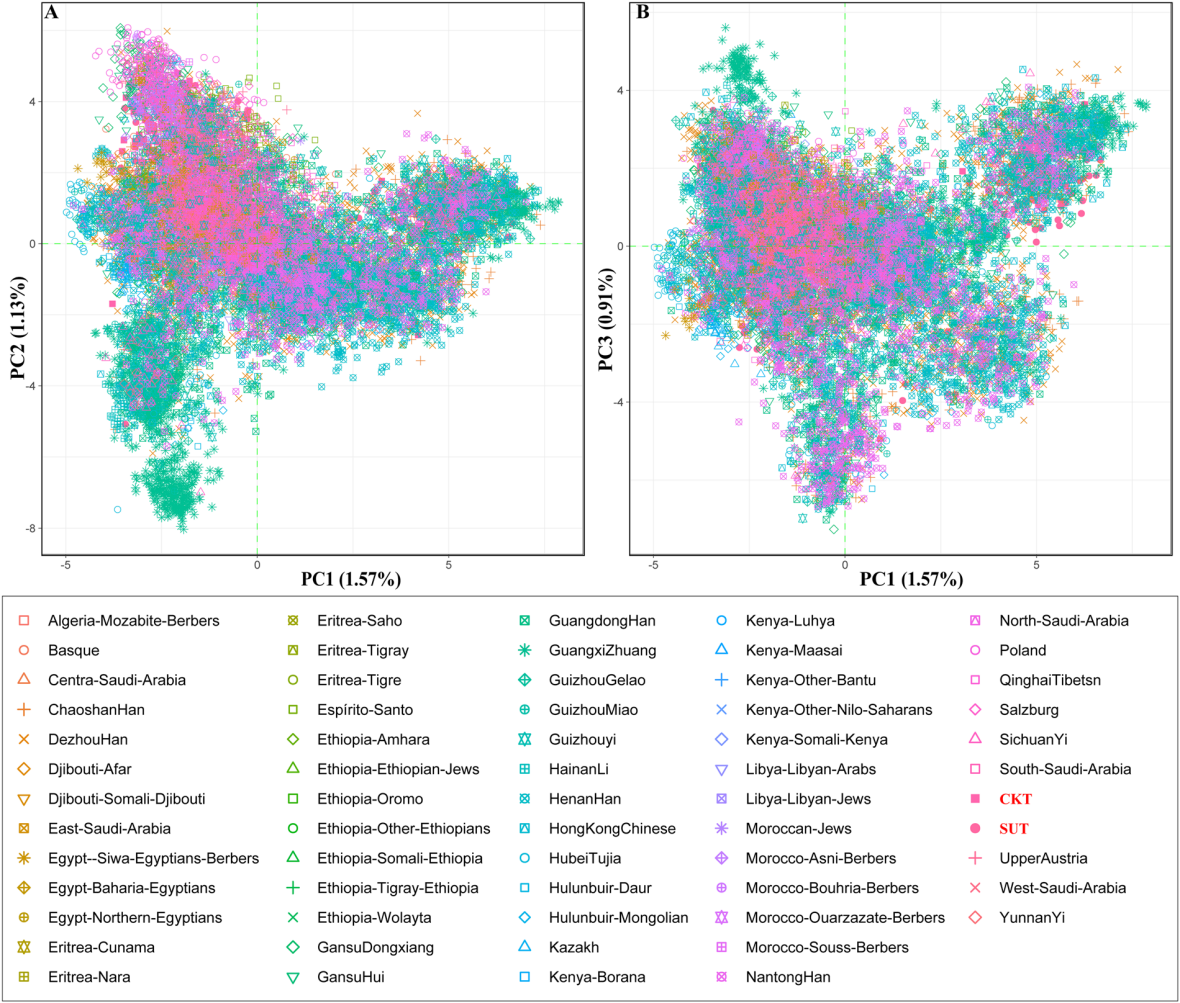
Principal component analysis among 63 worldwide populations.

**Figure 3:**
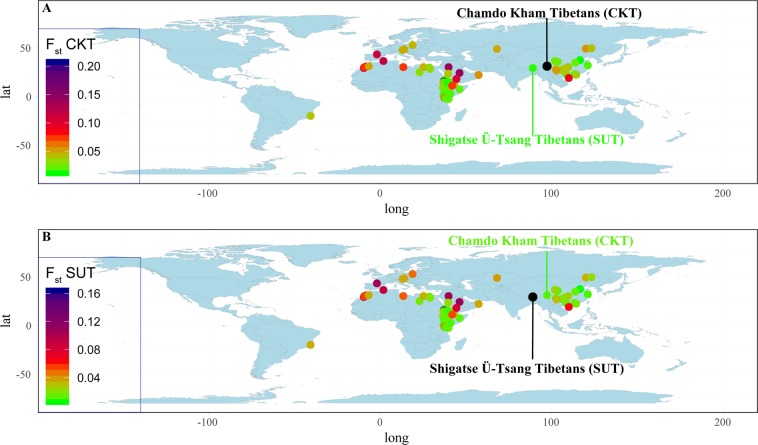
Heatmaps show the pairwise genetic distances between Tibetans and the other 61 reference populations.

To explore the population genetic relationship capitalizing the worldwide Y-chromosomal STR variations, we merged our data with the publicly available data from 27 Y-STRs from 15,880 individuals (Table S4). The top three principal components (PC) could explain 3.61% of the variation (Fig. 2). No clear population stratification was observed. This may be caused by the high proportion of shared Y-chromosome haplotypes in modern male populations. The pairwise Fst genetic distances among 63 populations were calculated and visualized as the heat map in Fig. S1. The Dezhou Han population harbors the smallest genetic distance between both CKT (0.0112) and SUT (0.0054). We can also identify the relatively small genetic distances between Tibetan and Northeast African populations (Fig. 3), which may be caused by the small sample number of these African populations. A phylogenetic relationship reconstruction based on the pairwise Fst genetic distances generally shows the distinct genetic affinity between African and Asian populations, but we also found confusing cluster patterns between Northeast African populations and others. Two newly studied Tibetan populations maintain a close phylogenetic relationship with Qinghai Tibetan populations (Fig. S2). To obtain a clearer genetic landscape of Tibetan populations and more adjacent populations with larger sample sizes, we focused on the genetic diversity in Asia and China.

### Overall genetic affinity between Tibetans and other Asian populations

**Figure 4:**
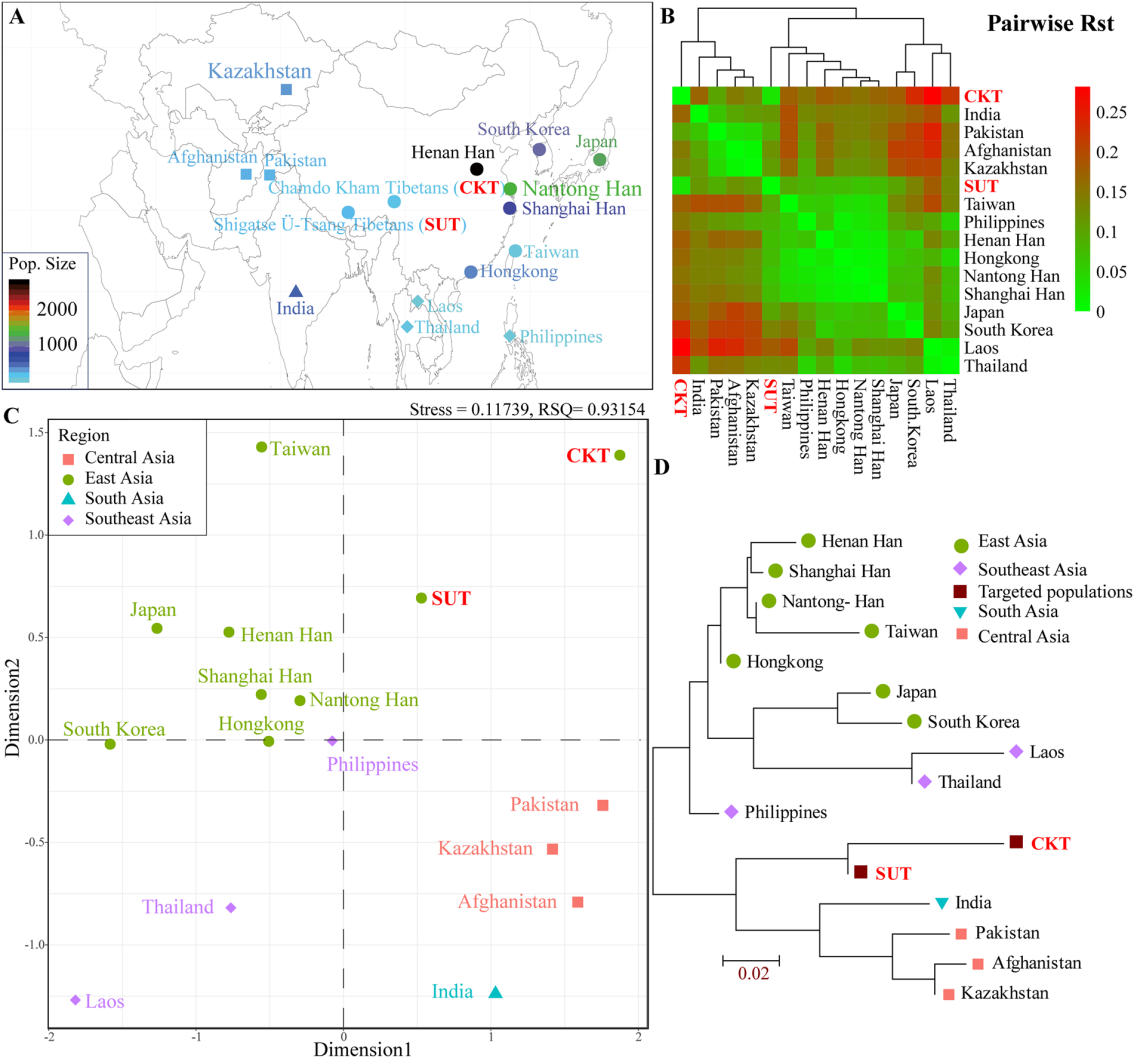
Genetic affinity among 16 Asian populations. (**A**) The map shows the geographic location and population size of the 16 Asian populations. The map was generated by free software R 3.3.2 (https://www.R-project.org/)^49^ using the ggrepel, ggplot2, maps and mapdata packages and then modified using Adobe Illustrator CS6 software. The R script is submitted in Supplementary Note 1. (**B**) The heatmap shows the pairwise genetic distance among 16 included populations. (**C**) Multidimensional scaling plots show the genetic similarities among Asian populations. (**D**) The phylogenetic tree shows the relationship among 16 groups.

To obtain a generally comprehensive picture of genetic architecture between two newly studied Tibetan populations and Asian populations, we first merged our haplotype data from 402 samples with previously published haplotype data from 9,577 samples in fourteen populations (Fig. 4A). Pairwise Rst standard genetic distances among sixteen populations were calculated and visualized in Fig. 4B. CKT was genetically closest to SUT (Rst = 0.029), followed by Central Asian populations of Pakistan (Rst = 0.1002), Kazakhstan (Rst = 0.1292) and Afghanistan (Rst = 0.1458). Shigatse Tibetans had the smallest genetic distance from CKT (Rst = 0.029), followed by the East Asian populations of Nantong Han (Rst = 0.0652), Henan Han (Rst = 0.0723) and Shanghai Han (Rst = 0.738). Genetic homogeneity and heterogeneity on the basis of the Rst distance matrix were further explored via multidimensional scaling plots (MDS). As shown in Fig. 4C, CKT and SUT maintain a relatively close relationship and are located in the first quadrant. East Asian populations and one group from the Philippines are clustered together and positioned in the second quadrant. The Southeast Asian populations from Thailand and Laos are localized in the third quadrant. Central Asian populations (Pakistan, Kazakhstan and Afghanistan) and one South Asian population from India are grouped in the fourth quadrant. Consistent patterns of genetic affinity among sixteen populations were reconstructed in the neighbor-joining tree (Fig. 4D). East Asian and Southeast Asian populations form one branch, and our newly studied populations combined with the Central Asian population and South Asian population form the other branch. In total, the genetic similarities observed in the Asian populations correspond to geographical divisions.

### Population structure and phylogenetic relationships among Chinese populations

**Figure 5:**
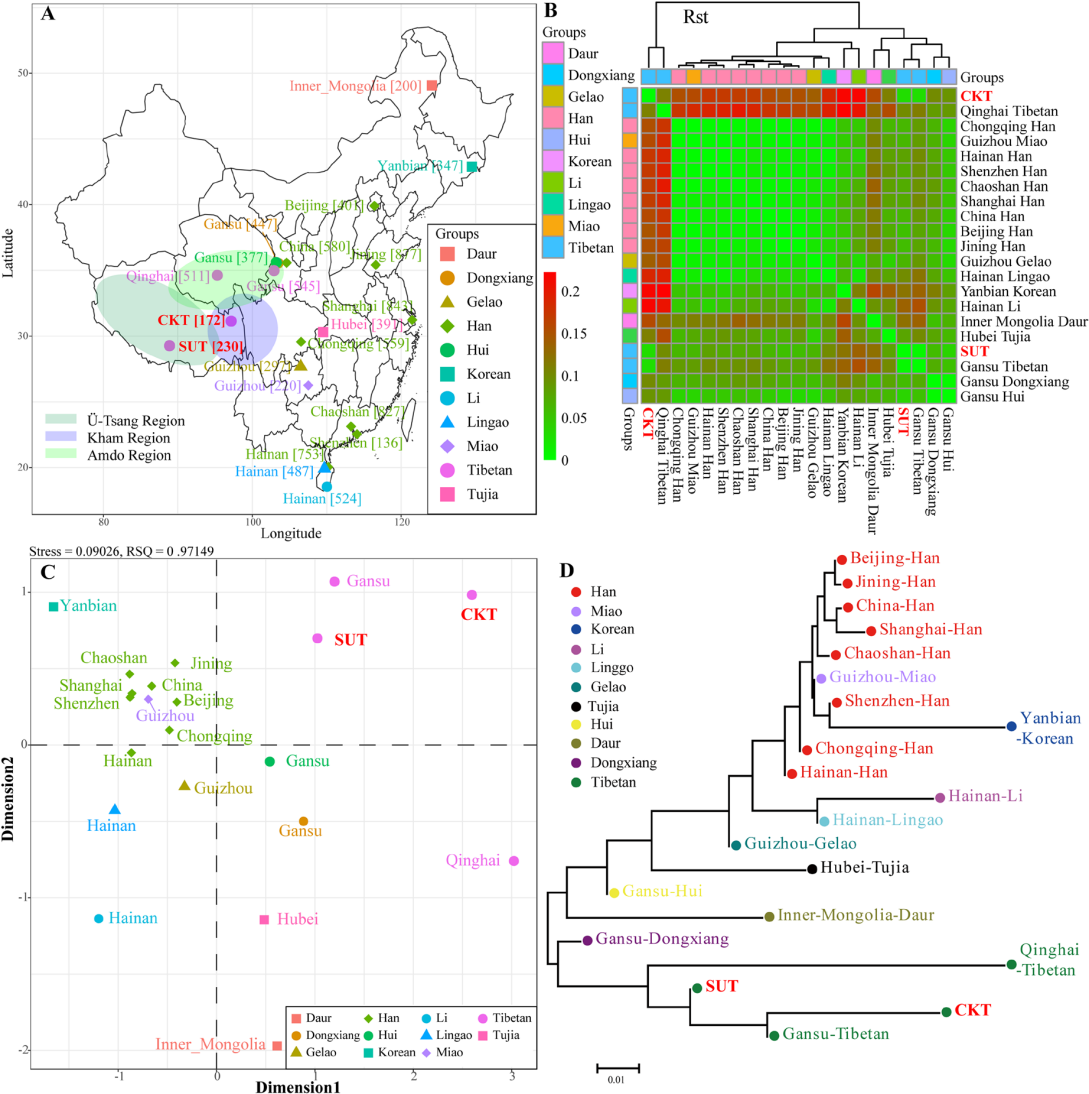
Genetic similarities and differences between two newly investigated Tibetan populations and 19 Chinese reference populations. (**A**) Geographical positions of 21 Chinese populations and the Kham, Ando and Ü-Tsang regions. The map was generated by free software R 3.3.2 (https://www.R-project.org/)^49^ with the scripts listed in Supplementary Note 2 and was then modified using Adobe Illustrator CS6 software. (**B**) The map displays the pairwise Rst standard genetic distance between SUT, CKT and 19 Chinese populations. (**C**) Genetic relationships were explored using two-dimensional scaling plots. (**D**) The phylogenetic relationship was investigated via a neighbor-joining algorithm.

Genetic affinity and population structure between CKT, SUT and Chinese reference populations^20,35–41^ were then evaluated using the pairwise genetic distances, MDS and phylogenetic relationship reconstruction (Fig. 5A). The pairwise Rst distances with corresponding p values are presented in Supplementary Table S5, and a heatmap of the pairwise genetic distance matrix is presented in Fig. 5B. Among the 21 Chinese populations, CKT has the closest genetic relationships with Gansu Ando Tibetans (Rst = 0.0281) and SUT (Rst = 0.0291) and has significantly distinct genetic relationships with the Hainan Li (Rst = 0.2174) and Yanbian Korean (Rst = 0.2174) populations, which has overall temperate genetic variances (average ± standard variance: 0.1478 ± 0.0511). Similar patterns of genetic variation and population relationships of SUT were observed with an average standard variance of 0.0726 ± 0.0318. Population genetic relationships are subsequently reconstructed using the MDS and neighbor-joining tree. As shown in Fig. 5C, MDS shows a genetic homogeneity among three Tibetan populations (Gansu Ando, Chamdo Kham and Shigatse Ü-Tsang), as well as genetic similarity within eight Han Chinese populations (Shanghai, Chaoshan, Hainan, Shenzhen, Beijing, Chongqing, Jining and China), Guizhou Gelao and Hainan Lingao. Significant genetic differences were observed among the Yanbian Korean, Gansu Hui, Hanna Li, Gansu Dongxiang, Qinghai Tibetan, Inner Mongolia Daur and Hubei Tujia populations. Two clusters of population relationship patterns were observed in the phylogenetic tree: four Tibetan populations and one Dongxiang population form one genetic affinity cluster, and other Chinese minorities and Han Chinese populations form the other cluster (Fig. 5D).

Genetic distance comparisons revealed that strong genetic affinity exists among Tibetans along different cultural backgrounds and geographical adjacent minorities (Gansu Dongxiang), as well as significant genetic differentiation between Tibetans and homogeneous Han Chinese populations. Significant genetic differences between Tibetan populations and other reference populations may be explicable by unique demographical history, genetic origin and divergence. Archeological evidence based on Paleolithic sites on the Tibetan Plateau suggested that anatomically modern humans resided in this region with the most extreme environmental conditions over 20 kya^2,44^. Tibetan matrilineal genetic evidence indicated that Tibetans are descended from Epipaleolithic and Neolithic immigrants from northern China^6^. Recently, a large number of genetic studies found that unique natural selection mechanisms have driven Tibetans to accumulate different genetic legacies, especially genetic mutations in EPAS1 and EGLN1^9,17^. Lu *et al*. found that Tibetans and the Han Chinese diverged more than 15 kya and found genetic coexistence and continuity between prehistorical highland-foragers (Paleolithic, Neolithic ages) and modern Tibetans^1^. Our current study provided a forensic reference database of Chinese Tibetan populations and general population genetic relationships between Chinese Tibetan populations and other global populations. Further genetic studies of the combined testing of modern people and ancient human remains are needed to provide clear and more accurate demographical and evolutionary histories of this Tibetan population adapted to high altitude.

## Conclusion

In summary, we investigated the distribution of Y-chromosome variations (27 Y-STRs) in two high-altitude populations (SUT and CKT). This highly polymorphic and informative PCR amplification system, especially with the included high-mutational markers, could potentially be useful for regional or national reference reconstruction for forensic paternity testing, missing person investigations and disaster victim identification. Population genetic analyses among worldwide and Asian and Chinese populations demonstrated that genetic affinity exists within the three diverse cultural Tibetans and adjacent ethnicities. Significant genetic distinctions exist between Tibetans and other geographically/ethnically distant populations.

## Materials and Methods

### Sample collection and DNA extraction

The sample collection protocol and design for this study were considered and approved by the Ethics Committee at the Institute of Forensic Medicine, West China School of Basic Medical Sciences & Forensic Medicine, Sichuan University (K2015008). A total of 402 blood stains were sampled from two groups on the Qinghai-Tibet Plateau: 172 Chamdo Kham male Tibetans residing in East Tibet and 230 Shigatse Ü-Tsang male Tibetans residing in West Tibet. All donors were unrelated healthy indigenous people and provided written informed consent. DNA was extracted using the PureLink Genomic DNA Mini Kit (Thermo Fisher Scientific, Waltham, MA, USA) and quantified via the Applied Biosystems 7500 Real-time PCR System (Thermo Fisher Scientific) using the Quantifiler Human DNA Quantification Kit (Thermo Fisher Scientific) following the manufacturer’s recommendations.

### DNA amplification and genotyping

Co-amplification of 27 Y-STRs (DYS576, DYS19, YGATAH4, DYS448, DYS391, DYS456, DYS390, DYS438, DYS392, DYS627, DYS460, DYS458, DYS449, DYS393, DYS518, DYS570, DYS437, DYS385, DYS389I, DYS635, DYS389II, DYS439, DYS481, DYF387S1 and DYS533) was performed using the Yfiler Plus PCR Amplification Kit (Thermo Fisher Scientific) on the ProFlex 3 × 32 PCR System (Thermo Fisher Scientific) according to the manufacturer’s instructions. Amplified DNA products were separated and detected using capillary electrophoresis on a 3500XL Genetic Analyzer (Thermo Fisher Scientific) with the LIZ-600 size standard and ABI Hi-Di^TM^ deionized formamide. Fragment nomenclature was conducted using GeneMapper1 IDX v.1.4 (Thermo Fisher Scientific).

### Statistical analysis

Haplotype and allele frequencies of Kham and Ü-Tsang Tibetans were calculated on the basis of the direct counting method. Gene diversity (GD), haplotype diversity (HD) and discrimination capacity (DC) were measured using the following formulas^26,45^:

1 $${\rm{GD}}=\frac{N}{N-1}(1-\sum {P}_{ai}^{2}),$$

2 $${\rm{HD}}=\frac{N}{N-1}(1-\sum {P}_{hi}^{2}),$$

3 $$MP=\sum {P}_{ai}^{2},$$

where *N* indicates the total number of samples of one population, *P*_*ai*_ indicates the allele frequency of *i* th allele in one locus, and *P*_*hi*_ denotes the haplotype frequency of the *i* th haplotype in one population. Pairwise Fst genetic distances between two studied Tibetan populations and 61 other worldwide populations (total of 15,880 raw genotypes) were calculated using Arlequin suite ver 3.5^46^. Principal component analysis based on the raw genotype data was conducted using the convenient online tool for STR Analysis for Forensics (STRAF)^47^. The samples included duplicates or triplicates, and null alleles were removed in the pairwise genetic distance evaluation. Pairwise genetic distances (Rst) and corresponding p values between our Tibetans and other adjacent relative populations with the same or different ethno-geographic origins were calculated using online tools in the YHRD database according to the online instructions in the YHRD datable (https://yhrd.org/amova). Finally, multidimensional scaling plot (MDS) and neighbor-joining tree were constructed on the basis of the Rst genetic matrixes using the YHRD online tool and Molecular Evolutionary Genetics Analysis Version 7.0 (Mega 7.0)^48^, respectively. The map used in this study was generated by free software R 3.3.2 (https://www.R-project.org/)^49^.

### Quality control

Control DNA 007 was used as the positive control and ddH_2_O (Thermo Fisher Scientific) was used as the negative control in each batch of amplification and genotyping. We performed our experiments in the laboratory in the Department of Forensic Genetics, West China School of Basic Medical Sciences & Forensic Medicine, Sichuan University, which is accredited by the China National Accreditation Service for Conformity Assessment (CNAS) and participated the quality control of the YHARD database. We strictly followed the recommendations published by the DNA Commission of the International Society for Forensic Genetics (ISFG)^50,51^. All the methods were carried out in accordance with the approved guidelines of Institute of Forensic Medicine, Sichuan University.

## Supplementary information

Supplementary Materials Figures S1-2 and Tables S1-S5
