# Supplementary material for: Genetic structure and forensic characteristics of Tibeto-Burman-speaking Ü-Tsang and Kham Tibetan Highlanders revealed by 27 Y-chromosomal STRs
Source: Sci Rep. 2019 May 23;9:7739. doi: 10.1038/s41598-019-44230-2 (PMC6533295; doi:10.1038/s41598-019-44230-2)
Supplement: Supplementary file 1 — Supplementary Materials Figures S1-2 and Tables S1-S5 [file 41598_2019_44230_MOESM1_ESM.pdf]

Supplementary Materials Figures S1-2 and Tables S1-S5

Genetic structure and forensic characteristics of Tibeto-Burman-speaking Ü-Tsang and Kham Tibetan Highlanders revealed by 27 Y-chromosomal STRs

Guanglin He[1, #], Zheng Wang[1, #], Yongdong Su[2, #], Xing Zou[1], Mengge Wang[1], Xu Chen[3], Bo Gao[4], Jing Liu[1], Shouyu Wang[1], Yiping Hou[1,*]

[1]Institute of Forensic Medicine, West China School of Basic Medical Sciences & Forensic Medicine, Sichuan University, Chengdu, Sichuan 610041, China

[2]Forensic Identification Center, Public Security Bureau of Tibet Tibetan Autonomous Region, Lhasa, Tibet Tibetan Autonomous Region 850000, China

[3]Department of Clinical Laboratory, the First People's Hospital of Liangshan Yi Autonomous Prefecture, Xichang, Sichuan 615000, China

[4]Yili Public Security Bureau, Yili, Xinjiang Uygur Autonomous Region 418000, China

[#]Guanglin He, Yongdong Su and Zheng Wang contributed equally to this work and should be considered as co-first authors.

[*]Corresponding author

Yiping Hou

Affiliation: Institute of Forensic Medicine, West China School of Basic Medical Sciences & Forensic Medicine, Sichuan University, Chengdu, China; Tel.: +86-28-85501549; Fax: +86-28-85501549;

E-mail: forensic@scu.edu.cn, profhou@yahoo.com.

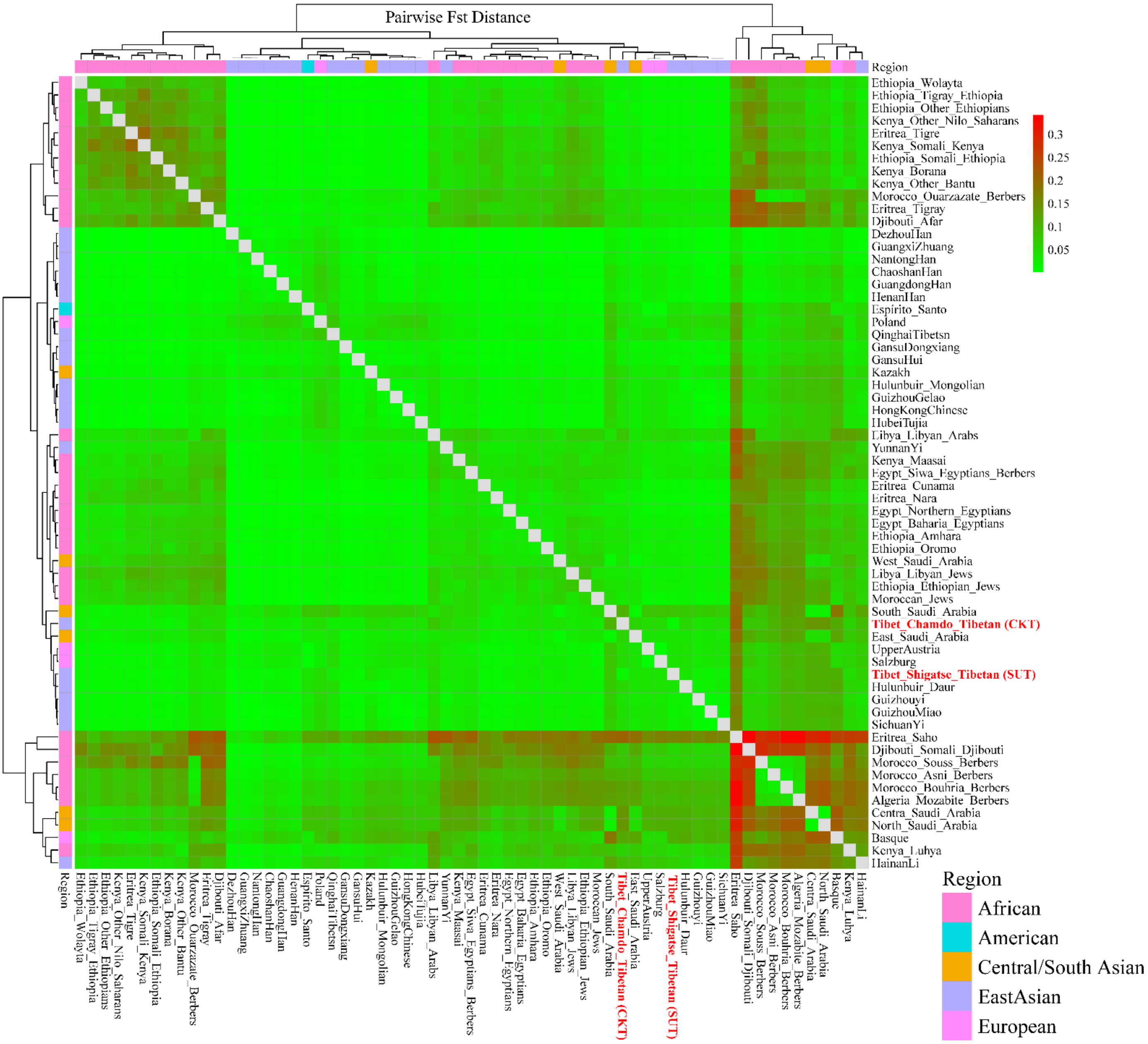

Figure S1. The pairwise Fst genetic distance between two studied Tibetans and other 61 worldwide reference populations.

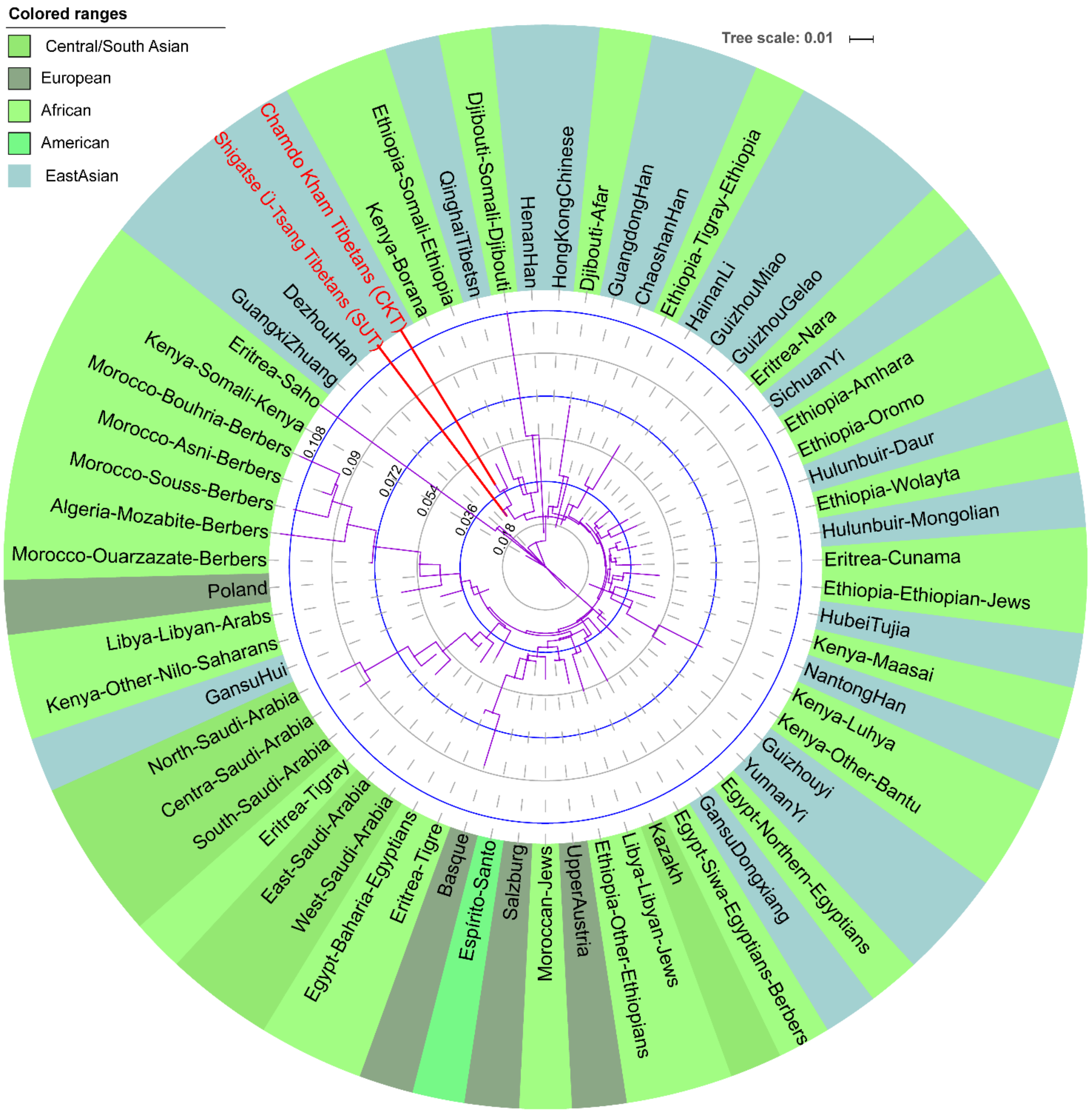

Figure S2. Phylogenetic relationships among 63 worldwide populations based on the Fst genetic distances.

Table S1. The genotype data and haplotype data of 230 Shigatse Ü-Tsang Tibetans and 172 Chamdo Kham Tibetans

ID	Pop	DYS576	DYS389I	DYS635	DYS389II	DYS627	DYS460	DYS458	DYS19	YGATAH4	DYS448	DYS391	DYS456	DYS390	DYS438	DYS392	DYS518	DYS570	DYS437	DYS385	DYS449	DYS393	DYS439	DYS481	DYF387S1	DYS533
CT001	Tibet_Chamdo_Tibetan	18	14	21	30	19	11	16	17	11	19	10	16	25	11	7	37	18	14	11,11	31	13	12	26	37,37	12
CT002	Tibet_Chamdo_Tibetan	18	12	20	27	17	10	17	14	12	20	10	15	23	11	15	37	18	16	13,20	34	12	12	23	36,37	11
CT003	Tibet_Chamdo_Tibetan	16	13	20	29	18	10	16	15	12	21	11	16	24	11	7	37	18	14	10,13	34	11	12	26	39,39	12
CT004	Tibet_Chamdo_Tibetan	16	12	21	28	19	12	20	15	11	19	11	14	24	10	10	37	20	14	16,16	32	12	11	32	37,37	12
CT005	Tibet_Chamdo_Tibetan	19	13	21	29	20	11	17	15	11	19	9	17	25	11	7	38	18	14	11,11	31	13	12	25	37,37	12
CT006	Tibet_Chamdo_Tibetan	16	14	21	30	20	11	16	15	11	19	10	16	25	11	7	39	20	14	11,11	33	13	11	25	37,38	12
CT007	Tibet_Chamdo_Tibetan	17	12	20	27	19	10	17	14	12	20	10	15	23	11	15	37	19	16	13,20	34	12	12	23	36,36	11
CT008	Tibet_Chamdo_Tibetan	18	12	20	27	18	10	17	14	12	20	10	14	23	11	15	34	18	15	13,20	32	12	12	23	36,37	11
CT009	Tibet_Chamdo_Tibetan	18	12	20	30	21	10	19	15	11	20	10	16	25	10	10	36	19	14	15,16	30	12	13	28	37,38	12
CT010	Tibet_Chamdo_Tibetan	18	13	21	29	19	11	15	16	11	19	10	16	25	11	7	39	19	14	11,11	31	13	12	27	37,38	12
CT011	Tibet_Chamdo_Tibetan	16	14	22	30	21	11	17	15	11	20	10	16	25	11	7	38	19	14	11,14	30	13	11	28	36,37	12
CT012	Tibet_Chamdo_Tibetan	18	12	21	27	19	10	17	14	12	20	10	14	23	11	15	35	19	16	13,20	32	12	12	23	36,37	11
CT013	Tibet_Chamdo_Tibetan	19	14	22	30	20	11	18	15	11	18	10	16	24	11	7	37	20	14	11,11	31	13	12	26	37,38	12
CT014	Tibet_Chamdo_Tibetan	18	15	21	31	20	11	16	15	10	19	10	16	27	11	7	37	20	14	11,11	31	13	13	25	38,39	11
CT015	Tibet_Chamdo_Tibetan	18	13	21	29	19	11	15	16	11	19	10	16	25	11	7	39	19	14	11,11	32	13	12	27	37,38	12
CT016	Tibet_Chamdo_Tibetan	16	14	22	30	21	11	17	15	11	19	10	16	25	11	7	38	19	14	11,14	29	13	11	27	35,37	12
CT017	Tibet_Chamdo_Tibetan	17	13	21	29	21	12	17	14	12	19	11	14	23	11	16	39	19	14	11,13	28	14	11	20	37,37	11
CT018	Tibet_Chamdo_Tibetan	16	13	22	29	22	10	17	15	11	19	10	16	25	11	7	38	21	14	11,15	31	13	11	29	36,37	12
CT019	Tibet_Chamdo_Tibetan	18	14	21	30	19	10	16	15	10	19	10	16	25	11	7	37	19	14	11,12	31	13	12	25	37,38	11
CT020	Tibet_Chamdo_Tibetan	19	12	21	28	19	9	18	15	12	20	10	15	23	11	12	42	16	15	12,15	33	13	12	22	35,37	12
CT021	Tibet_Chamdo_Tibetan	19	14	22	30	20	13	19	15	11	18	10	16	25	11	7	39	22	14	11,11	30	13	11	25	37,37	12
CT022	Tibet_Chamdo_Tibetan	18	14	21	30	19	11	16	15	10	19	10	16	25	11	7	37	19	14	11,11	31	13	12	25	36,38	11
CT023	Tibet_Chamdo_Tibetan	20	14	22	30	20	11	17	16	11	18	10	15	25	11	7	39	19	14	11,11	31	13	12	27	37,38	12
CT024	Tibet_Chamdo_Tibetan	16	14	21	30	21	11	17	15	11	20	10	16	25	11	7	38	19	14	11,14	30	13	11	28	36,37	12
CT025	Tibet_Chamdo_Tibetan	17	14	21	30	21	10	16	15	11	19	10	16	25	11	7	39	19	14	11,14	31	13	11	28	36,37	12
CT026	Tibet_Chamdo_Tibetan	17	13	21	29	19	11	15	16	11	19	10	16	25	11	7	39	19	14	11,11	31	13	12	27	37,38	12
CT027	Tibet_Chamdo_Tibetan	17	14	22	31	23	11	15	15	13	19	11	15	23	11	14	37	19	14	12,12	29	13	11	24	37,37	12
CT028	Tibet_Chamdo_Tibetan	16	12	20	28	19	11	19	14	11	19	9	14	24	10	10	36	20	14	17,17	31	12	11	29	37,38	11
CT029	Tibet_Chamdo_Tibetan	16	12	20	28	19	12	19	14	11	19	9	14	24	10	10	36	20	14	17,17	31	12	11	29	37,38	11
CT030	Tibet_Chamdo_Tibetan	18	12	20	27	17	10	17	14	12	20	10	15	23	11	15	36	18	16	13,20	33	12	12	23	36,37	11
CT031	Tibet_Chamdo_Tibetan	18	15	22	32	23	11	15	15	13	19	11	15	23	11	14	37	19	14	12,12	29	13	11	24	37,37	12

CT032	Tibet_Chamdo_Tibetan	17	14	23	30	19	12	17	15	11	19	11	15	25	11	7	38	19	14	11,11	33	13	12	25	37,38	12
CT033	Tibet_Chamdo_Tibetan	17	12	20	27	18	10	17	14	12	20	10	16	23	11	15	37	19	15	13,21	34	12	12	23	36,37	11
CT034	Tibet_Chamdo_Tibetan	17	12	20	27	18	10	17	14	12	20	10	16	23	11	15	37	19	15	13,21	34	12	12	23	36,36	11
CT035	Tibet_Chamdo_Tibetan	19	14	22	32	22	10	15	14	12	19	10	17	23	11	14	37	17	14	11,12	29	13	11	24	39,39	12
CT036	Tibet_Chamdo_Tibetan	18	12	20	26	17	9	17	13	12	20	10	15	23	11	15	36	17	15	13,19	33	12	13	23	36,36	11
CT037	Tibet_Chamdo_Tibetan	19	12	21	27	18	10	17	14	11	20	10	15	23	11	14	34	18	15	13,20	34	12	12	23	36,37	11
CT038	Tibet_Chamdo_Tibetan	18	14	21	30	20	10	17	15	11	19	10	15	25	11	7	39	19	14	11,11	31	13	12	25	37,38	12
CT039	Tibet_Chamdo_Tibetan	19	14	21	30	19	10	16	15	10	19	10	16	25	11	7	37	20	14	11,11	31	13	11	25	37,39	11
CT040	Tibet_Chamdo_Tibetan	18	14	21	30	20	12	17	15	11	18	10	16	24	11	7	39	18	14	11,11	30	13	12	26	37,38	11
CT041	Tibet_Chamdo_Tibetan	18	14	21	30	21	11	16	15	11	19	10	16	25	11	7	39	19	14	10,11	32	13	11	25	37,38	12
CT042	Tibet_Chamdo_Tibetan	18	13	23	29	19	10	17	15	11	19	10	16	25	11	7	38	19	14	11,11	31	13	12	25	36,38	12
CT043	Tibet_Chamdo_Tibetan	17	14	22	30	22	10	16	15	11	19	10	15	26	11	7	38	18	14	11,14	30	13	11	27	37,37	12
CT044	Tibet_Chamdo_Tibetan	19	14	21	30	20	11	17	15	11	18	10	16	25	11	7	38	20	14	11,11	30	13	12	25	37,37	12
CT045	Tibet_Chamdo_Tibetan	19	14	21	30	20	11	17	15	11	18	10	16	25	11	7	38	20	14	11,11	30	13	12	25	37,38	12
CT046	Tibet_Chamdo_Tibetan	18	12	20	27	19	10	17	14	12	20	10	15	23	11	15	36	18	16	13,21	33	12	12	23	36,37	11
CT047	Tibet_Chamdo_Tibetan	18	12	20	27	19	10	17	14	12	20	10	15	23	11	15	36	18	16	13,21	33	12	12	23	36,37	12
CT048	Tibet_Chamdo_Tibetan	16	14	21	30	21	11	17	15	11	19	10	16	25	11	7	38	20	14	11,14	31	13	11	28	36,37	12
CT049	Tibet_Chamdo_Tibetan	18	12	21	27	19	10	17	14	12	20	10	14	23	11	15	35	18	16	13,20	32	12	12	23	36,37	11
CT050	Tibet_Chamdo_Tibetan	19	13	21	29	19	11	17	14	12	19	11	15	22	10	14	36	18	14	12,13	27	13	12	22	37,37	12
CT051	Tibet_Chamdo_Tibetan	17	12	20	27	19	10	17	14	12	20	10	15	23	11	14	38	18	14	13,19	34	12	12	22	37,37	11
CT052	Tibet_Chamdo_Tibetan	17	13	21	30	21	11	18	15	11	18	10	15	23	11	7	37	18	14	11,14	30	13	13	28	37,37	11
CT053	Tibet_Chamdo_Tibetan	17	13	21	30	21	11	18	15	11	18	10	15	23	11	7	37	18	14	11,14	30	13	13	28	37,37	12
CT054	Tibet_Chamdo_Tibetan	16	14	21	30	21	11	17	15	11	20	10	16	25	11	7	38	19	14	11,14	30	13	11	28	36,37	12
CT055	Tibet_Chamdo_Tibetan	18	14	22	31	23	11	15	15	13	19	11	15	23	11	14	37	19	14	12,12	29	13	11	24	37,37	12
CT056	Tibet_Chamdo_Tibetan	17	12	20	27	19	10	18	14	12	20	10	15	23	11	15	36	19	15	13,20	34	12	12	23	36,36	11
CT057	Tibet_Chamdo_Tibetan	18	14	21	29	21	11	16	15	11	18	10	16	25	11	7	38	20	14	11,11	29	13	12	25	37,38	12
CT058	Tibet_Chamdo_Tibetan	16	15	21	31	20	11	16	15	12	19	10	15	26	11	7	39	20	14	11,11	32	13	12	25	38,38	12
CT059	Tibet_Chamdo_Tibetan	17	12	20	28	19	12	20	15	11	19	10	14	24	10	10	35	20	14	16,17	32	12	11	30	38,38	11
CT060	Tibet_Chamdo_Tibetan	19	12	23	28	19	10	19	15	11	19	10	14	25	10	13	39	20	14	12,20	30	12	12	25	36,38	11
CT061	Tibet_Chamdo_Tibetan	17	12	20	28	19	11	21	15	11	19	10	14	24	10	10	35	20	14	16,17	32	12	11	30	37,38	11
CT062	Tibet_Chamdo_Tibetan	19	14	21	30	20	11	15	14	12	19	11	15	23	11	14	35	19	14	11,12	30	13	11	23	35,35	11
CT063	Tibet_Chamdo_Tibetan	17	12	20	28	19	11	20	15	11	19	10	14	24	10	10	35	20	14	16,17	32	12	11	30	37,38	11
CT064	Tibet_Chamdo_Tibetan	19	12	23	28	20	10	19	15	11	19	10	14	25	10	13	39	20	14	12,20	30	12	12	25	36,38	11

CT065	Tibet_Chamdo_Tibetan	21	13	24	29	21	11	17	14	12	18	10	16	22	11	14	33	19	14	12,12	27	13	12	22	37,37	12
CT066	Tibet_Chamdo_Tibetan	18	13	21	28	19	9	17	14	12	20	10	15	23	11	14	35	18	15	14,19	32	12	12	23	35,38	11
CT067	Tibet_Chamdo_Tibetan	22	13	24	30	20	11	17	14	12	18	11	16	22	10	14	33	19	14	12,12	27	13	12	22	37,37	12
CT068	Tibet_Chamdo_Tibetan	17	13	21	30	19	11	16	15	11	19	10	15	24	11	7	39	20	14	11,15	28	13	12	27	37,38	12
CT069	Tibet_Chamdo_Tibetan	18	14	21	30	22	11	17	15	11	19	10	15	26	11	7	40	20	15	11,14	31	13	13	29	36,37	12
CT070	Tibet_Chamdo_Tibetan	18	13	24	29	24	10	15	14	12	19	11	14	23	11	14	36	16	14	11,12	28	13	12	22	38,38	12
CT071	Tibet_Chamdo_Tibetan	20	12	19	28	22	10	17	15	12	19	10	15	24	10	12	35	16	15	12,16	33	12	12	22	36,39	11
CT072	Tibet_Chamdo_Tibetan	18	12	20	29	22	11	19	15	11	19	10	15	25	10	10	35	20	14	16,16	31	12	11	27	35,38	12
CT073	Tibet_Chamdo_Tibetan	16	14	21	31	22	11	15	15	13	19	11	15	23	11	14	37	18	14	12,12	28	13	11	24	37,37	12
CT074	Tibet_Chamdo_Tibetan	18	13	21	29	21	10	18	16	11	17	10	15	24	10	11	42	19	14	14,18	29	12	10	28	36,37	11
CT075	Tibet_Chamdo_Tibetan	17	13	21	29	20	10	17	15	12	19	11	16	25	11	7	38	19	14	11,11	33	13	12	25	37,39	12
CT076	Tibet_Chamdo_Tibetan	17	12	20	28	22	11	19	15	11	19	10	15	25	10	10	35	21	14	16,16	32	12	11	27	35,38	12
CT077	Tibet_Chamdo_Tibetan	19	14	21	30	21	11	15	14	12	19	11	15	23	11	14	35	19	14	11,12	30	13	11	23	35,35	11
CT078	Tibet_Chamdo_Tibetan	18	14	21	30	20	10	16	15	10	19	10	15	26	11	7	37	20	14	11,11	31	13	11	25	37,38	11
CT079	Tibet_Chamdo_Tibetan	18	13	21	29	20	10	16	16	11	19	10	15	25	11	7	39	19	14	11,11	31	14	12	25	37,37	12
CT080	Tibet_Chamdo_Tibetan	18	13	22	29	20	11	16	16	11	17	11	15	25	10	13	42	16	14	17,19	29	14	13	22	36,37	10
CT081	Tibet_Chamdo_Tibetan	17	13	21	30	19	11	17	15	11	19	10	15	24	11	7	39	20	14	11,15	28	13	12	27	37,38	12
CT082	Tibet_Chamdo_Tibetan	17	14	22	30	21	12	17	15	11	19	11	16	25	11	7	38	18	14	11,11	35	13	12	19	37,39	12
CT083	Tibet_Chamdo_Tibetan	20	14	21	30	19	11	16	15	10	19	10	16	26	11	7	36	18	14	11,11	31	13	12	25	37,39	11
CT084	Tibet_Chamdo_Tibetan	18	13	22	29	19	11	16	15	11	20	10	15	25	11	7	40	18	14	11,11	31	13	12	25	37,38	12
CT085	Tibet_Chamdo_Tibetan	18	13	22	29	20	11	18	15	11	19	10	17	25	11	7	38	20	14	11,11	31	13	13	25	37,38	12
CT086	Tibet_Chamdo_Tibetan	19	14	21	30	21	11	16	15	11	19	10	15	25	11	7	41	17	15	11,15	30	13	12	30	36,37	12
CT087	Tibet_Chamdo_Tibetan	16	14	21	30	21	11	17	15	11	19	10	16	25	11	7	38	19	14	11,14	31	13	11	28	36,37	12
CT088	Tibet_Chamdo_Tibetan	18	12	20	27	17	10	16	14	12	20	10	15	23	11	15	37	18	16	13,20	34	12	11	23	37,37	11
CT089	Tibet_Chamdo_Tibetan	18	12	19	29	23	10	15	15	12	18	11	16	23	10	14	38	17	14	13,13	31	13	11	25	36,41	11
CT090	Tibet_Chamdo_Tibetan	18	12	20	27	18	10	16	14	12	20	10	15	23	11	15	37	18	16	13,20	32	12	12	23	36,37	11
CT091	Tibet_Chamdo_Tibetan	18	12	20	27	18	10	17	14	12	20	10	15	22	11	15	37	17	16	13,20	33	12	12	24	35,37	11
CT092	Tibet_Chamdo_Tibetan	18	14	21	30	20	11	16	15	11	19	10	16	25	11	7	38	19	14	11,11	32	13	12	25	37,38	12
CT093	Tibet_Chamdo_Tibetan	18	15	21	31	19	11	16	15	11	19	11	16	25	11	7	37	20	14	11,11	31	13	13	25	37,38	12
CT094	Tibet_Chamdo_Tibetan	19	14	21	30	19	11	16	15	10	19	10	16	26	11	7	37	20	14	11,11	30	13	12	25	37,39	11
CT095	Tibet_Chamdo_Tibetan	17	12	20	30	18	10	20	15	11	20	10	16	25	10	10	35	19	14	15,16	30	12	12	28	36,38	12
CT096	Tibet_Chamdo_Tibetan	17	12	20	28	21	10	17	14	12	20	10	15	23	11	14	36	19	15	13,15	31	12	12	23	34,36	11
CT097	Tibet_Chamdo_Tibetan	17	13	21	30	20	12	19	15	11	19	10	15	24	11	7	36	18	14	11,14	32	13	12	28	37,37	12

CT098	Tibet_Chamdo_Tibetan	18	14	21	30	20	12	16	16	11	19	10	16	25	11	7	38	19	14	11,11	31	13	12	25	36,37	12
CT099	Tibet_Chamdo_Tibetan	17	14	22	30	20	11	16	15	11	19	11	16	25	11	7	37	19	14	11,11	33	13	12	25	37,38	12
CT100	Tibet_Chamdo_Tibetan	17	12	20	28	18	9	18	14	12	20	10	14	24	11	14	35	18	15	13,18	33	12	12	23	35,37	12
CT101	Tibet_Chamdo_Tibetan	19	13	23	29	19	11	17	15	11	20	10	15	25	11	7	41	18	14	11,11	32	13	12	24	36,38	12
CT102	Tibet_Chamdo_Tibetan	19	14	23	31	22	11	16	14	12	19	11	17	23	11	15	36	19	14	11,12	29	13	11	24	37,37	12
CT103	Tibet_Chamdo_Tibetan	19	12	19	27	22	9	18	15	13	19	10	16	23	11	12	38	16	15	12,17	32	13	12	22	35,39	13
CT104	Tibet_Chamdo_Tibetan	17	15	21	31	21	11	17	15	11	19	10	16	25	11	7	38	18	14	11,14	30	13	11	28	36,38	12
CT105	Tibet_Chamdo_Tibetan	18	14	21	30	20	11	16	15	11	19	10	15	25	11	7	38	19	14	11,11	31	13	13	25	37,38	12
CT106	Tibet_Chamdo_Tibetan	18	14	22	32	22	11	16	14	13	19	11	16	23	11	14	37	19	14	11,12	29	13	11	24	38,38	12
CT107	Tibet_Chamdo_Tibetan	17	14	22	30	21	11	17	15	11	19	10	15	25	11	7	40	21	14	11,13	31	13	12	28	36,37	12
CT108	Tibet_Chamdo_Tibetan	18	12	20	27	18	10	18	14	12	20	10	15	23	11	15	37	18	16	13,19	32	12	12	23	36,37	11
CT109	Tibet_Chamdo_Tibetan	16	14	20	30	19	11	16	15	11	18	11	15	23	10	14	37	22	14	10,12	30	13	10	19	35,36	13
CT110	Tibet_Chamdo_Tibetan	17	15	21	31	21	11	15	15	11	19	10	16	25	11	7	38	19	14	11,14	30	13	11	28	36,38	12
CT111	Tibet_Chamdo_Tibetan	18	12	20	26	18	10	15	14	12	20	10	15	23	11	14	36	18	15	14,20	32	12	13	23	35,37	12
CT112	Tibet_Chamdo_Tibetan	17	12	21	26	20	11	18	17	12	20	11	14	26	10	13	41	21	13	12,19	29	12	12	24	36,38	11
CT113	Tibet_Chamdo_Tibetan	18	14	22	32	22	11	16	14	13	19	11	16	23	11	14	37	19	14	11,12	29	13	11	24	37,37	12
CT114	Tibet_Chamdo_Tibetan	18	12	22	29	22	11	20	15	11	19	10	15	25	10	10	36	17	14	15,16	30	13	11	28	35,37	12
CT115	Tibet_Chamdo_Tibetan	18	12	20	27	18	10	17	14	12	20	10	16	23	11	15	37	18	15	13,20	33	12	12	23	37,37	11
CT116	Tibet_Chamdo_Tibetan	16	14	23	31	19	12	15	14	11	19	10	15	23	10	14	39	19	14	11,12	29	13	11	22	36,36	13
CT117	Tibet_Chamdo_Tibetan	16	14	20	30	21	11	17	15	11	19	10	16	25	11	7	38	20	14	11,14	29	13	12	28	36,37	12
CT118	Tibet_Chamdo_Tibetan	16	14	20	30	19	12	16	15	11	18	11	15	23	10	14	37	22	14	10,12	30	13	10	19	35,36	13
CT119	Tibet_Chamdo_Tibetan	18	12	20	29	22	11	20	15	11	20	10	16	25	10	10	35	19	14	15,16	29	12	12	28	38,39	12
CT120	Tibet_Chamdo_Tibetan	17	14	23	31	22	11	15	14	12	19	11	18	23	11	14	36	19	14	11,12	29	13	11	24	38,38	12
CT121	Tibet_Chamdo_Tibetan	16	13	22	29	22	11	16	15	11	19	11	16	25	11	7	37	19	14	11,14	30	13	12	28	37,38	12
CT122	Tibet_Chamdo_Tibetan	12	15	21	31	21	11	17	15	11	19	10	16	25	11	7	38	19	14	11,14	30	13	11	28	36,38	12
CT123	Tibet_Chamdo_Tibetan	18	12	20	27	18	9	17	14	12	20	10	16	23	11	14	36	17	15	13,21	32	12	13	23	36,36	11
CT124	Tibet_Chamdo_Tibetan	20	14	22	29	20	11	17	14	12	19	11	15	23	10	14	36	17	14	12,13	27	13	11	22	37,37	12
CT125	Tibet_Chamdo_Tibetan	17	12	20	28	17	9	17	14	12	20	10	15	23	11	14	35	16	15	14,14	32	12	13	24	36,37	11
CT126	Tibet_Chamdo_Tibetan	17	14	21	30	19	11	17	15	11	19	10	15	25	11	7	37	19	14	11,11	32	14	12	26	37,39	11
CT127	Tibet_Chamdo_Tibetan	18	12	20	27	18	10	17	14	12	20	10	15	23	11	14	36	18	16	13,20	34	12	12	23	36,37	11
CT128	Tibet_Chamdo_Tibetan	18	12	20	28	18	9	18	14	11	20	10	16	23	11	14	35	19	15	13,20	32	12	12	23	37,38	12
CT129	Tibet_Chamdo_Tibetan	16	14	20	30	21	11	17	15	11	19	10	16	25	11	7	38	20	14	11,14	29	13	12	28	36,37	12
CT130	Tibet_Chamdo_Tibetan	19	12	20	26	18	10	17	13	12	20	10	15	23	11	14	36	17	15	13,19	32	12	12	23	35,36	11

CT131	Tibet_Chamdo_Tibetan	16	14	21	30	21	11	17	15	11	19	10	16	25	11	7	38	18	14	11,14	30	13	11	28	36,37	12
CT132	Tibet_Chamdo_Tibetan	16	14	21	30	21	11	17	15	11	19	10	16	25	11	7	38	19	14	11,14	28	13	11	29	36,37	12
CT133	Tibet_Chamdo_Tibetan	16	14	21	30	22	11	17	15	11	19	10	16	25	11	7	39	19	14	11,14	30	13	12	28	36,37	12
CT134	Tibet_Chamdo_Tibetan	17	13	22	29	20	11	16	15	11	19	10	15	25	11	7	38	19	14	11,11	32	13	11	25	36,37	12
CT135	Tibet_Chamdo_Tibetan	17	15	21	31	21	11	17	15	11	19	10	16	25	11	7	38	19	14	11,14	30	13	11	28	36,38	12
CT136	Tibet_Chamdo_Tibetan	19	14	21	30	20	10	17	14	12	19	11	15	22	10	14	36	18	14	13,13	28	13	13	23	37,38	12
CT137	Tibet_Chamdo_Tibetan	18	13	22	30	21	10	16	15	12	20	10	17	23	11	7	42	21	14	11,13	31	12	13	26	39,39	12
CT138	Tibet_Chamdo_Tibetan	20	13	22	29	19	10	17	15	11	17	10	14	24	10	11	40	18	14	14,19	30	12	10	26	37,37	12
CT139	Tibet_Chamdo_Tibetan	17	14	24	30	22	9	16	16	11	20	10	16	24	11	7	36	19	14	13,15	30	12	11	26	37,37	11
CT140	Tibet_Chamdo_Tibetan	21	12	20	28	19	10	18	15	12	20	10	16	23	11	14	35	18	16	13,20	32	12	13	23	35,37	11
CT141	Tibet_Chamdo_Tibetan	16	14	21	31	21	12	17	15	11	19	10	15	25	11	7	39	18	14	11,14	30	13	11	29	36,37	12
CT142	Tibet_Chamdo_Tibetan	18	14	23	32	23	11	16	14	12	19	11	16	23	11	14	36	20	14	11,12	29	13	11	24	38,38	12
CT143	Tibet_Chamdo_Tibetan	18	13	20	28	18	10	17	14	12	20	10	15	23	11	15	37	18	16	13,19	33	12	12	23	36,37	11
CT144	Tibet_Chamdo_Tibetan	16	14	21	30	21	11	16	15	11	19	10	16	25	11	7	38	19	14	11,14	29	13	11	29	37,37	12
CT145	Tibet_Chamdo_Tibetan	17	14	21	30	20	11	16	16	11	20	10	16	25	11	7	38	19	14	11,11	31	13	12	25	37,38	12
CT146	Tibet_Chamdo_Tibetan	16	14	21	30	21	11	17	15	11	19	10	16	25	11	7	38	18	14	11,14	29	13	12	29	37,37	12
CT147	Tibet_Chamdo_Tibetan	16	14	23	30	22	11	16	14	12	19	11	16	23	11	14	37	19	14	11,12	29	13	11	24	38,38	12
CT148	Tibet_Chamdo_Tibetan	18	13	24	29	23	10	14	15	12	21	9	15	24	9	11	39	19	14	12,17	31	12	11	22	37,39	12
CT149	Tibet_Chamdo_Tibetan	18	13	20	28	18	10	17	14	12	20	10	15	23	11	15	37	18	16	13,20	33	12	12	23	36,37	11
CT150	Tibet_Chamdo_Tibetan	16	14	23	30	22	11	16	14	12	19	11	16	23	11	14	37	19	14	11,12	29	13	11	24	38,38	12
CT151	Tibet_Chamdo_Tibetan	17	14	21	30	19	11	17	15	11	19	10	15	25	11	7	37	19	14	11,11	31	13	12	25	37,38	11
CT152	Tibet_Chamdo_Tibetan	18	14	21	30	19	11	16	15	11	19	10	15	25	11	7	37	20	14	11,12	32	13	12	25	37,38	11
CT153	Tibet_Chamdo_Tibetan	17	14	22	30	19	11	16	16	10	19	10	16	26	11	7	37	19	15	11,12	31	13	12	26	37,38	12
CT154	Tibet_Chamdo_Tibetan	17	14	22	30	19	11	16	16	10	19	10	16	26	11	7	37	19	15	11,12	31	13	12	26	37,38	11
CT155	Tibet_Chamdo_Tibetan	19	12	21	28	19	9	17	15	12	20	10	15	24	11	14	35	17	15	13,19	32	13	12	23	35,38	11
CT156	Tibet_Chamdo_Tibetan	18	12	20	27	18	10	17	14	12	20	10	15	23	11	15	36	18	15	13,20	34	12	12	23	36,36	11
CT157	Tibet_Chamdo_Tibetan	16	14	22	30	22	10	16	15	11	19	10	15	25	11	7	37	18	14	11,14	30	13	11	27	37,37	12
CT158	Tibet_Chamdo_Tibetan	16	14	22	30	22	9	16	15	11	19	10	15	25	11	7	37	18	14	11,14	30	13	11	27	37,37	12
CT159	Tibet_Chamdo_Tibetan	18	14	21	30	21	11	16	15	10	19	10	16	26	11	7	37	19	14	11,11	31	13	13	25	37,38	11
CT160	Tibet_Chamdo_Tibetan	16	14	20	31	19	11	17	16	14	22	11	16	24	11	7	34	17	14	11,14	34	11	12	26	39,39	12
CT161	Tibet_Chamdo_Tibetan	17	13	21	30	16	9	18	15	12	20	10	15	23	11	15	36	19	15	13,17	32	12	12	23	36,38	11
CT162	Tibet_Chamdo_Tibetan	18	11	21	27	18	11	19	15	12	20	10	16	23	11	14	36	19	16	13,18	32	12	13	23	35,37	11
CT163	Tibet_Chamdo_Tibetan	18	13	23	30	22	12	15	14	12	19	11	16	23	11	14	35	20	14	11,12	29	13	11	24	37,37	12

CT164	Tibet_Chamdo_Tibetan	17	13	21	30	22	11	16	15	11	19	10	17	25	11	7	41	21	14	11,14	30	13	12	30	37,38	12
CT165	Tibet_Chamdo_Tibetan	19	14	21	30	20	11	17	15	11	18	11	16	23	11	7	39	19	14	11,11	30	13	12	25	37,37	12
CT166	Tibet_Chamdo_Tibetan	19	14	22	29	20	12	15	13	12	19	11	16	23	11	14	36	20	14	11,12	30	13	11	24	38,38	12
CT167	Tibet_Chamdo_Tibetan	19	13	24	29	21	11	17	15	13	19	11	15	24	12	13	37	17	15	11,14	30	13	12	22	35,37	13
CT168	Tibet_Chamdo_Tibetan	16	12	20	26	20	9	19	14	12	20	11	15	23	11	14	37	17	14	13,20	33	12	13	23	35,38	11
CT169	Tibet_Chamdo_Tibetan	18	14	21	29	19	9	16	14	12	20	10	15	23	11	14	35	18	14	13,18	31	12	12	23	36,38	11
CT170	Tibet_Chamdo_Tibetan	19	12	19	27	21	9	15	15	12	19	10	15	23	11	12	38	16	15	12,17	32	14	11	22	34,39	12
CT171	Tibet_Chamdo_Tibetan	20	13	21	29	20	10	17	16	11	17	11	14	24	10	11	39	17	14	14,19	29	12	10	27	37,37	12
CT172	Tibet_Chamdo_Tibetan	16	14	21	30	21	10	16	16	11	19	10	16	25	11	7	38	19	14	11,14	30	13	11	28	36,36	11
ST001	Tibet_Shigatse_Tibetan	18	13	21	29	22	11	18	14	11	19	10	15	24	10	11	38	18	15	13,16	28	12	11	23	37,39	12
ST002	Tibet_Shigatse_Tibetan	17	14	22	30	20	11	15	15	11	21	10	15	23	10	11	41	16	14	11,18	33	13	11	26	38,39	12
ST003	Tibet_Shigatse_Tibetan	19	13	21	29	20	11	16	15	11	19	10	14	24	11	7	38	19	14	11,14	30	13	13	28	38,40	12
ST004	Tibet_Shigatse_Tibetan	19	12	20	28	18	9	18	14	12	20	10	15	24	11	14	36	18	15	14,18	32	12	14	23	35,39	11
ST005	Tibet_Shigatse_Tibetan	19	12	21	28	22	10	17	14	11	17	10	15	24	10	11	41	18	14	14,19	30	12	10	27	36,36	11
ST006	Tibet_Shigatse_Tibetan	19	13	21	29	21	10	18	14	11	16	11	15	24	10	11	39	19	14	15,19	29	12	10	26	35,38	11
ST007	Tibet_Shigatse_Tibetan	18	12	20	28	19	10	19	15	12	19	10	15	24	11	14	35	18	16	12,19	31	12	12	23	35,38	11
ST008	Tibet_Shigatse_Tibetan	18	14	21	30	21	10	17	15	11	18	11	15	22	10	13	38	20	15	11,14	30	14	11	27	35,37	10
ST009	Tibet_Shigatse_Tibetan	19	12	20	27	19	8	20	14	12	20	10	16	23	11	14	35	17	15	13,16	32	12	11	23	35,38	11
ST010	Tibet_Shigatse_Tibetan	16	12	21	29	23	11	21	15	11	19	10	15	27	10	10	35	20	14	17,18	31	12	11	25	36,36	12
ST011	Tibet_Shigatse_Tibetan	17	12	20	30	25	11	20	15	11	19	10	15	27	10	10	35	20	14	17,18	29	12	11	28	37,37	12
ST012	Tibet_Shigatse_Tibetan	18	12	22	28	18	10	18	15	12	20	10	16	23	11	14	36	20	16	13,19	36	12	12	23	35,37	11
ST013	Tibet_Shigatse_Tibetan	17	14	21	30	20	11	17	15	10	18	10	17	25	11	7	40	21	14	11,11	29	13	12	25	37,38	12
ST014	Tibet_Shigatse_Tibetan	16	12	20	28	19	11	19	15	11	19	10	14	24	10	10	36	19	14	16,17	31	13	11	30	37,38	11
ST015	Tibet_Shigatse_Tibetan	18	13	21	29	22	10	15	14	11	17	10	15	24	10	11	40	19	14	14,14	30	12	10	26	36,39	12
ST016	Tibet_Shigatse_Tibetan	20	14	22	30	22	11	15	14	11	18	10	15	24	10	14	40	18	14	13,19	29	14	13	23	36,39	10
ST017	Tibet_Shigatse_Tibetan	18	12	20	28	18	10	18	15	12	20	10	15	23	11	14	35	20	14	13,17	29	12	12	23	36,38	11
ST018	Tibet_Shigatse_Tibetan	17	14	24	30	19	11	15	14	12	19	11	16	24	11	14	37	19	14	11,12	28	13	12	22	37,37	12
ST019	Tibet_Shigatse_Tibetan	19	12	20	27	19	9	17	14	13	20	10	15	24	11	14	36	17	15	13,13	34	12	12	23	37,37	11
ST020	Tibet_Shigatse_Tibetan	16	13	22	30	23	11	21	15	11	19	10	15	24	10	10	37	17	14	16,17	29	12	11	27	36,37	12
ST021	Tibet_Shigatse_Tibetan	16	13	20	30	20	11	16	15	11	19	10	14	25	11	7	39	19	14	11,15	33	13	12	20	36,36	12
ST022	Tibet_Shigatse_Tibetan	18	12	20	29	19	9	16	14	12	20	10	15	23	11	14	36	18	14	13,20	34	12	12	23	35,38	11
ST023	Tibet_Shigatse_Tibetan	20	12	20	28	18	9	18	13	12	20	10	15	25	11	14	36	18	15	14,18	31	12	12	24	35,41	12
ST024	Tibet_Shigatse_Tibetan	17	12	21	28	21	11	15	15	11	21	10	15	23	10	11	37	16	14	11,17	30	13	11	26	38,39	12

ST025	Tibet_Shigatse_Tibetan	18	13	21	29	22	10	17	16	11	17	10	15	23	10	11	37	18	14	14,18	30	12	10	24	37,37	12
ST026	Tibet_Shigatse_Tibetan	18	12	20	29	22	11	22	15	11	19	11	15	27	10	10	35	17	14	16,16	30	12	11	28	36,36	12
ST027	Tibet_Shigatse_Tibetan	17	13	21	30	19	11	16	15	11	19	10	15	24	11	7	39	21	14	11,14	28	13	12	25	38,38	12
ST028	Tibet_Shigatse_Tibetan	18	14	21	30	20	12	16	15	11	20	10	17	24	11	7	38	20	14	11,11	31	13	13	25	37,37	12
ST029	Tibet_Shigatse_Tibetan	18	12	21	29	18	9	18	14	12	20	10	15	22	11	14	36	18	16	13,17	32	12	13	22	34,37	12
ST030	Tibet_Shigatse_Tibetan	17	12	21	28	19	9	17	15	12	20	10	15	23	11	14	36	19	14	13,17	31	12	12	24	36,37	11
ST031	Tibet_Shigatse_Tibetan	18	14	21	30	20	11	17	15	11	18	10	15	23	11	7	39	20	14	11,11	31	13	11	25	38,39	12
ST032	Tibet_Shigatse_Tibetan	17	15	22	31	19	11	15	14	12	19	11	15	24	11	14	39	18	14	12,12	28	13	12	22	37,37	12
ST033	Tibet_Shigatse_Tibetan	17	12	20	28	20	9	17	14	12	20	10	15	23	11	14	35	18	15	13,18	32	12	13	23	35,39	11
ST034	Tibet_Shigatse_Tibetan	18	14	21	30	19	11	16	15	11	19	10	16	25	11	7	37	18	14	11,11	31	13	12	25	37,38	11
ST035	Tibet_Shigatse_Tibetan	18	13	21	30	20	11	17	16	11	19	10	16	25	11	7	38	18	14	11,11	31	13	13	25	37,38	12
ST036	Tibet_Shigatse_Tibetan	17	14	23	30	18	11	15	14	12	18	11	15	23	11	15	37	18	15	12,12	28	13	12	22	37,37	12
ST037	Tibet_Shigatse_Tibetan	18,19	14	21	30	21	11	16	15	11	20	10	15	24	11	7	40	18,19	14	11,15	31	14	12	27	36,43	12
ST038	Tibet_Shigatse_Tibetan	19	12	20	28	20	9	18	14	12	20	10	15	23	11	14	37	18	15	13,18	32	12	12	23	35,38	11
ST039	Tibet_Shigatse_Tibetan	19	12	21	28	20	9	16	14	12	20	10	16	23	11	14	35	19	15	13,19	31	12	13	23	35,36	11
ST040	Tibet_Shigatse_Tibetan	18	14	21	30	20	11	16	15	10	19	10	16	26	11	7	39	20	14	11,11	31	13	12	25	37,38	11
ST041	Tibet_Shigatse_Tibetan	19	13	21	30	24	11	20	15	11	19	10	14	24	9	11	38	16	15	13,15	29	13	12	25	38,39	12
ST042	Tibet_Shigatse_Tibetan	18	12	20	29	22	11	22	15	11	19	10	15	27	10	10	35	19	14	17,17	31	12	11	28	36,36	12
ST043	Tibet_Shigatse_Tibetan	18	13	21	29	20	11	17	15	11	19	10	16	23	11	7	37	19	14	11,11	30	13	12	25	37,38	12
ST044	Tibet_Shigatse_Tibetan	16	12	20	29	19	11	20	15	11	19	10	14	24	11	10	36	20	14	16,18	31	12	11	29	37,39	11
ST045	Tibet_Shigatse_Tibetan	17	12	21	29	20	11	16	15	10	21	11	15	22	10	11	37	21	16	17,17	31	14	11	22	38,39	9
ST046	Tibet_Shigatse_Tibetan	20	12	20	28	18	9	19	14	12	20	10	15	24	11	14	34	17	15	12,19	31	12	13	23	40,41	11
ST047	Tibet_Shigatse_Tibetan	18	14	21	30	22	11	17	16	11	19	11	16	25	11	7	38	19	14	11,11	31	13	12	25	37,37	12
ST048	Tibet_Shigatse_Tibetan	18	12	20	28	19	9	20	14	11	20	10	15	23	11	14	37	20	15	13,18	32	12	12	23	35,38	11
ST049	Tibet_Shigatse_Tibetan	19	12	21	28	19	9	17	15	12	20	10	15	23	11	14	37	20	15	12,19	31	12	12	23	35,38	11
ST050	Tibet_Shigatse_Tibetan	21	12	20	28	18	9	17	14	12	20	10	14	24	11	14	36	20	15	14,18	33	12	12	23	35,37	11
ST051	Tibet_Shigatse_Tibetan	19	12	20	27	19	9	19	14	12	20	11	15	24	11	14	35	18	15	13,18	31	12	12	23	35,36	11
ST052	Tibet_Shigatse_Tibetan	17	12	20	28	18	9	17	14	12	20	10	15	22	9	14	35	17	15	14,19	32	12	12	23	36,37	11
ST053	Tibet_Shigatse_Tibetan	18	14	21	30	18	11	17	15	11	19	10	15	25	11	7	37	20	14	11,11	33	13	12	26	37,37	12
ST054	Tibet_Shigatse_Tibetan	19	14	22	30	20	10	17	15	12	18	10	16	23	11	7	38	19	14	11,11	29	13	12	25	37,37	12
ST055	Tibet_Shigatse_Tibetan	19	14	22	30	21	12	15	14	12	19	11	16	24	11	14	36	20	14	11,12	29	13	12	19	37,37	12
ST056	Tibet_Shigatse_Tibetan	16	12	23	29	23	10	20	17	12	21	10	14	25	10	13	39	18	14	14,18	27	12	11	24	37,38	11
ST057	Tibet_Shigatse_Tibetan	17	12	21	28	19	9	18	15	12	19	10	15	23	11	15	36	19	16	13,17	32	12	13	23	35,38	11

ST058	Tibet_Shigatse_Tibetan	18	13	22	29	18	11	13	17	11	19	11	15	25	11	7	42	18	14	11,11	32	12	12	25	37,38	12
ST059	Tibet_Shigatse_Tibetan	18	13	20	29	19	11	18	15	12	19	10	16	24	11	14	36	18	16	12,18	30	12	12	23	34,38	11
ST060	Tibet_Shigatse_Tibetan	19	12	21	28	23	10	20	17	11	20	10	15	24	10	13	37	19	15	14,20	31	12	12	26	36,37	11
ST061	Tibet_Shigatse_Tibetan	19	12	20	26	18	10	18	14	12	20	10	15	23	11	14	36	18	15	13,19	33	12	12	23	36,36	11
ST062	Tibet_Shigatse_Tibetan	17	12	20	28	18	9	17	14	13	21	10	15	23	11	14	35	18	15	14,19	32	12	12	23	35,37	12
ST063	Tibet_Shigatse_Tibetan	18	14	22	30	19	11	17	15	11	18	10	16	23	11	7	39	20	14	11,11	31	13	12	26	37,38	12
ST064	Tibet_Shigatse_Tibetan	17	12	22	25	18	9	17	15	12	20	10	14	23	11	14	37	19	15	13,17	34	12	13	24	36,38	11
ST065	Tibet_Shigatse_Tibetan	17	13	20	31	19	11	16	15	11	19	10	15	24	11	7	38	19	14	12,14	28	13	11	28	37,38	12
ST066	Tibet_Shigatse_Tibetan	19	12	21	28	19	9	16	14	12	20	10	15	24	11	14	35	17	15	13,19	30	12	12	23	35,38	11
ST067	Tibet_Shigatse_Tibetan	17	14	21	30	22	11	17	16	11	19	11	16	25	11	7	38	20	14	11,11	31	13	12	25	37,37	12
ST068	Tibet_Shigatse_Tibetan	19	12	21	28	17	9	18	15	11	20	10	15	23	11	14	35	19	14	13,19	31	12	12	22	35,37	11
ST069	Tibet_Shigatse_Tibetan	18	14	22	30	19	11	16	15	11	19	10	15	27	11	7	40	18	14	11,11	35	13	12	25	37,38	11
ST070	Tibet_Shigatse_Tibetan	18	12	21	27	21	9	15	15	12	20	10	15	22	11	14	35	18	16	13,18	33	12	13	22	35,37	11
ST071	Tibet_Shigatse_Tibetan	19	12	20	27	18	9	18	14	13	21	10	16	23	11	14	35	18	15	14,19	34	12	12	23	36,38	11
ST072	Tibet_Shigatse_Tibetan	19	12	21	28	19	9	18	14	12	21	10	15	23	11	14	34	18	15	13,19	32	12	14	23	35,40	11
ST073	Tibet_Shigatse_Tibetan	18	12	20	29	19	9	18	14	12	20	10	15	23	11	14	37	18	15	14,19	34	12	13	23	35,37	11
ST074	Tibet_Shigatse_Tibetan	18	13	21	29	19	10	16	15	11	19	10	14	24	11	7	37	19	14	11,14	31	13	12	27	38,40	13
ST075	Tibet_Shigatse_Tibetan	17	12	21	30	18	9	18	15	12	20	10	15	23	11	14	35	19	15	13,18	31	13	13	23	34,37	11
ST076	Tibet_Shigatse_Tibetan	17	14	21	30	20	10	16	15	11	19	10	14	25	11	7	38	19	14	11,11	32	13	12	24	37,37	12
ST077	Tibet_Shigatse_Tibetan	18	13	24	31	23	9	16	16	11	21	10	16	24	11	7	37	21	14	12,15	32	12	11	26	37,37	11
ST078	Tibet_Shigatse_Tibetan	19	14	22	31	22	11	16	14	12	19	11	16	23	11	14	36	19	14	12,12	30	13	11	24	38,38	12
ST079	Tibet_Shigatse_Tibetan	17	12	19	28	19	10	15	15	12	18	10	17	23	10	14	37	18	14	13,13	32	13	12	26	36,37	11
ST080	Tibet_Shigatse_Tibetan	16	14	24	30	22	10	17	14	11	19	10	15	19	10	13	36	16	15	12,13	32	13	13	25	35,36	13
ST081	Tibet_Shigatse_Tibetan	19	14	23	31	21	11	17	15,17	10	20	9	15	24	10	11	41	17	14	12,12	31	13	11	27	39,40	11
ST082	Tibet_Shigatse_Tibetan	19	14	21	30	19	11	16	17	11	19	10	15	24	11	7	37	19	14	11,11	32	14	12	25	37,37	11
ST083	Tibet_Shigatse_Tibetan	17	12	20	28	18	10	16	14	12	20	10	15	23	11	15	35	17	15	13,18	34	12	13	23	35,36	11
ST084	Tibet_Shigatse_Tibetan	18	14	21	31	22	11	15	14	13	19	11	16	23	11	14	36	20	14	11,12	29	13	12	24	38,38	12
ST085	Tibet_Shigatse_Tibetan	18	13	23	29	21	9	15	16	12	21	10	18	23	10	7	36	20	14	12,15	32	12	10	26	36,37	11
ST086	Tibet_Shigatse_Tibetan	16	14	18	31	21	10	17	15	10	19	10	15	22	10	11	37	19	14	14,19	32	15	11	24	39,39	12
ST087	Tibet_Shigatse_Tibetan	17	14	21	31	18	11	18	16	12	20	10	15	24	11	7	38	16	14	12,13	31	12	12	23	38,38	12
ST088	Tibet_Shigatse_Tibetan	18	13	21	29	20	11	17	16	11	19	10	16	25	11	7	38	18	14	11,11	31	13	13	25	37,38	12
ST089	Tibet_Shigatse_Tibetan	18	12	20	28	17	9	18	14	13	20	10	15	22	11	15	36	18	15	13,20	32	12	12	23	35,38	11
ST090	Tibet_Shigatse_Tibetan	16	12	20	28	18	9	19	15	12	20	10	15	23	11	13	36	18	16	13,17	34	12	14	22	35,37	11

ST091 Tibet_Shigatse_Tibetan	17	12	20	28	18	9	18	14	12	20	10	15	24	12	14	37	17	15	13,20	31	13	12	23	35,38	11
ST092 Tibet_Shigatse_Tibetan	16	13	21	29	22	11	15	17	11	19	10	17	24	11	7	36	19	14	11,14	31	13	13	28	37,38	12
ST093 Tibet_Shigatse_Tibetan	17	12	21	29	18	9	18	16	12	20	10	15	23	11	14	35	18	15	14,18	33	12	13	23	34,37	11
ST094 Tibet_Shigatse_Tibetan	18	14	22	30	19	10	17	15	11	19	10	16	25	11	7	38	18	14	11,11	33	13	12	26	38,39	12
ST095 Tibet_Shigatse_Tibetan	19	12	20	28	19	9	17	14	12	20	10	15	23	11	15	36	19	15	13,18	34	12	12	23	37,38	11
ST096 Tibet_Shigatse_Tibetan	18	12	20	28	19	9	17	14	12	20	11	15	23	11	14	37	18	15	14,18	33	12	13	23	36,38	11
ST097 Tibet_Shigatse_Tibetan	18	14	21	30	20	12	17	15	11	18	10	16	23	11	7	39	20	14	11,11	30	13	13	25	37,39	12
ST098 Tibet_Shigatse_Tibetan	18	13	21	29	19	11	15	15	10	19	10	16	26	11	7	37	19	14	11,11	31	14	12	25	37,38	11
ST099 Tibet_Shigatse_Tibetan	18	13	24	32	23	9	17	16	11	21	10	16	24	11	7	38	20	14	12,15	32	12	11	25	37,38	11
ST100 Tibet_Shigatse_Tibetan	18	12	20	28	15	10	16	14	12	20	10	15	25	11	14	34	16	15	12,19	32	12	12	23	35,41	11
ST101 Tibet_Shigatse_Tibetan	18	14	22	30	19	11	16	15	11	19	10	15	25	11	7	41	18	14	11,11	34	13	12	25	37,38	11
ST102 Tibet_Shigatse_Tibetan	18	12	21	28	19	9	18	14	12	21	10	15	23	11	14	34	18	15	13,19	32	12	14	23	35,40	11
ST103 Tibet_Shigatse_Tibetan	18	13	21	29	20	11	16	15	11	19	10	16	25	11	7	38	20	14	11,11	31	13	11	25	37,38	12
ST104 Tibet_Shigatse_Tibetan	20	13	21	29	20	11	16	15	11	19	10	14	24	11	7	38	19	14	11,15	30	13	13	29	38,40	12
ST105 Tibet_Shigatse_Tibetan	19	14	22	30	22	12	15	13	12	19	11	16	23	11	14	37	20	14	11,12	30	13	11	24	38,38	12
ST106 Tibet_Shigatse_Tibetan	17	12	20	27	18	9	17	14	13	20	10	15	23	11	14	35	18	15	14,19	32	12	12	24	35,37	12
ST107 Tibet_Shigatse_Tibetan	18	13	22	29	19	10	18	15	11	17	10	14	24	10	11	41	17	14	14,18	31	12	10	26	36,36	12
ST108 Tibet_Shigatse_Tibetan	18	12	21	28	19	11	17	15	12	19	10	15	23	11	14	36	18	15	13,18	32	12	12	23	37,37	11
ST109 Tibet_Shigatse_Tibetan	15	12	20	28	18	9	18	15	12	20	10	15	23	11	13	36	18	14	13,17	35	12	13	22	35,37	11
ST110 Tibet_Shigatse_Tibetan	20	12	20	28	18	9	17	15	12	21	10	15	25	11	14	35	17	15	13,19	32	12	12	23	35,38	11
ST111 Tibet_Shigatse_Tibetan	18	14	21	31	19.2	11	16	15	10	19	10	16	26	11	7	37	18	14	11,11	31	13	12	25	37,37	11
ST112 Tibet_Shigatse_Tibetan	19	14	22	31	22	11	16	14	12	19	11	15	23	11	14	37	19	14	11,12	30	13	11	24	38,38	12
ST113 Tibet_Shigatse_Tibetan	18	14	22	31	22	12	16	13	10	19	10	14	22	12	15	40	18	14	15,16	31	14	12	25	34,36	12
ST114 Tibet_Shigatse_Tibetan	18	13	22	29	19	10	19	15	11	17	10	14	24	10	11	41	17	14	14,18	31	12	10	26	36,36	12
ST115 Tibet_Shigatse_Tibetan	19	12	19	28	21	9	18	15	14	19	11	15	23	10	12	36	16	15	12,16	36	12	11	24	35,39	11
ST116 Tibet_Shigatse_Tibetan	19	13	23	29	20	11	17	15	11	17	10	15	24	10	11	37	18	14	14,20	30	12	10	25	37,37	12
ST117 Tibet_Shigatse_Tibetan	16	14	21	32	20	10	15	14	12	19	11	16	23	12	14	36	19	14	11,13	27	13	11	22	37,37	12
ST118 Tibet_Shigatse_Tibetan	16	14	21	30	19	11	16	15	10	19	10	16	26	11	7	37	20	14	11,11	32	13	12	25	37,38	11
ST119 Tibet_Shigatse_Tibetan	18	12	19	29	21	9	16	15	12	19	10	15	23	10	12	37	16	15	12,16	33	13	12	22	34,40	12
ST120 Tibet_Shigatse_Tibetan	18	12	19	29	22	9	16	15	12	19	10	15	23	10	12	36	16	15	12,16	33	13	12	22	34,40	12
ST121 Tibet_Shigatse_Tibetan	20	14	22	30	21	10	16	13	10	19	9	14	24	12	14	37.2	20	14	15,21	30	14	11	24	37,37	12
ST122 Tibet_Shigatse_Tibetan	18	12	21	27	18	10	17	14	11	20	10	15	23	11	14	36	18	15	13,21	33	12	12	23	36,37	11
ST123 Tibet_Shigatse_Tibetan	19	12	21	27	18	10	17	14	11	20	10	15	23	11	14	36	18	15	13,21	33	12	12	23	36,37	11

ST124	Tibet_Shigatse_Tibetan	16	14	24	30	19	11	16	14	13	19	10	15	24	11	14	38	17	14	11,12	29	13	12	22	37,37	12
ST125	Tibet_Shigatse_Tibetan	19	12	20	28	19	9	18	14	12	20	10	16	25	11	14	34	19	15	13,19	32	12	13	24	35,38	11
ST126	Tibet_Shigatse_Tibetan	18	13	21	29	21	10	18	15	11	17	10	15	25	10	11	43	20	14	14,18	28	12	10	28	35,36	11
ST127	Tibet_Shigatse_Tibetan	16	14	21	29	24	11	18	14	12	19	10	13	23	10	14	38	20	14	11,13	29	14	10	20	36,36	11
ST128	Tibet_Shigatse_Tibetan	20	13	22	29	20	11	17	15	11	20	10	15	25	11	7	40	18	14	11,12	33	13	14	25	37,39	12
ST129	Tibet_Shigatse_Tibetan	18	13	22	29	20	11	18	15	11	20	10	15	25	11	7	38	21	14	11,11	32	13	12	26	37,38	12
ST130	Tibet_Shigatse_Tibetan	19	12	20	27	22	11	17	15	12	19	11	16	22	10	13	38	17	15	12,16	32	12	12	22	36,39	11
ST131	Tibet_Shigatse_Tibetan	19	13	23	30	21	10	17	17	10	18	10	15	25	10	13	41	15	14	14,18	27	14	11	23	36,37	10
ST132	Tibet_Shigatse_Tibetan	19	12	19	28	21	10	15	15	12	18	10	17	23	10	14	40	18	14	13,14	32	13	11	25	38,41	11
ST133	Tibet_Shigatse_Tibetan	16	14	21	31	22	9	17	15	12	21	10	13	17	10	12	43	20	16	15,20	33	12	13	23	34,40	11
ST134	Tibet_Shigatse_Tibetan	17	12	21	27	20	10	17	16	12	20	8	14	24	10	13	38	21	14	12,21	28	12	11	25	36,37	11
ST135	Tibet_Shigatse_Tibetan	19	13	20	30	23	9	16	15	11	20	10	15	24	10	13	39	20	15	13,23	32	13	10	24	38,39	11
ST136	Tibet_Shigatse_Tibetan	18	12	20	28	23	11	21	16	11	19	10	15	25	10	10	35	20	14	16,16	32	12	11	31	35,39	13
ST137	Tibet_Shigatse_Tibetan	19	14	22	30	23	12	17	13	10	19	9	16	24	12	14	37.2	17	14	16,22	31	13	11	24	38,38	12
ST138	Tibet_Shigatse_Tibetan	18	12	23	28	21	10	18	16	12	18	10	14	25	10	13	38	17	14	11,21	30	12	11	24	35,39	12
ST139	Tibet_Shigatse_Tibetan	19	13	22	29	21	10	17	13	10	19	9	16	24	12	14	37.2	19	14	15,21	30	14	12	24	39,39	12
ST140	Tibet_Shigatse_Tibetan	18	12	24	28	24	10	17	14	12	18	10	15	23	10	14	37	17	14	12,13	27	13	10	22	38,38	13
ST141	Tibet_Shigatse_Tibetan	18	15	21	31	20	11	17	15	11	18	10	16	23	11	7	38	19	14	11,11	31	14	12	25	37,38	12
ST142	Tibet_Shigatse_Tibetan	17	13	22	30	22	11	15	14	12	19	11	15	23	11	14	36	19	14	11,12	30	13	11	24	38,38	12
ST143	Tibet_Shigatse_Tibetan	19	14	21	30	18	9	17	15	13	21	11	17	24	11	7	36	18	14	11,13	34	11	12	26	38,40	12
ST144	Tibet_Shigatse_Tibetan	16	14	22	30	24	11	15	14	12	19	11	14	23	10	14	40	20	14	11,13	29	14	10	21	36,36	11
ST145	Tibet_Shigatse_Tibetan	18	14	22	30	19	11	17	15	11	19	10	15	24	11	7	38	19	14	11,12	33	13	13	26	37,39	12
ST146	Tibet_Shigatse_Tibetan	18	12	20	28	19	9	20	13	12	20	10	15	23	11	14	37	18	14	13,19	32	12	11	23	35,38	11
ST147	Tibet_Shigatse_Tibetan	19	12	19	29	20	10	18	15	13	19	10	15	23	10	12	36	18	15	12,17	32	12	11	23	35,39	11
ST148	Tibet_Shigatse_Tibetan	18	13	21	30	21	11	15	15	11	19	10	17	25	11	7	36	19	14	11,11	31	13	12	25	37,38	12
ST149	Tibet_Shigatse_Tibetan	18	14	20	31	19	11	16	15	11	19	10	15	25	11	7	38	20	14	11,11	31	13	12	25	37,38	11
ST150	Tibet_Shigatse_Tibetan	16	14	22	30	24	11	16	14	12	19	11	14	23	10	14	40	20	14	11,13	29	14	10	21	36,36	11
ST151	Tibet_Shigatse_Tibetan	16	14	21	30	24	11	16	14	12	19	11	14	23	10	14	40	20	14	11,13	29	14	10	21	36,36	11
ST152	Tibet_Shigatse_Tibetan	16	14	22	30	24	11	16	14	12	19	11	14	23	10	14	40	20	14	11,13	29	14	10	21	37,37	11
ST153	Tibet_Shigatse_Tibetan	18	12	20	30	23	11	21	15	11	19	10	14	27	10	10	35	19	14	17,17	30	12	11	28	36,36	12
ST154	Tibet_Shigatse_Tibetan	19	14	21	30	20	11	16	15	11	19	10	16	25	11	7	38	19	14	11,11	31	13	11	25	37,38	11
ST155	Tibet_Shigatse_Tibetan	19	14	24	30	19	10	15	14	12	19	11	16	23	11	14	36	17	14	11,13	29	13	12	22	35,35	12
ST156	Tibet_Shigatse_Tibetan	19	14	22	31	25	11	17	14	10	19	9	15	24	12	14	37.2	19	14	17,22	32	14	11	24	39,39	12

ST157	Tibet_Shigatse_Tibetan	17	13	21	29	20	10	17	15	11	19	11	16	25	11	7	38	20	14	11,11	33	13	12	24	37,38	12
ST158	Tibet_Shigatse_Tibetan	18	12	23	29	19	9	17	13	12	19	10	15	25	10	13	41	19	14	12,12	33	13	13	23	36,40	11
ST159	Tibet_Shigatse_Tibetan	17	14	21	30	19	11	16	14	11	19	10	16	25	11	7	37	20	14	11,11	30	13	12	25	38,38	11
ST160	Tibet_Shigatse_Tibetan	18	13	21	28	23	11	17	15	12	19	11	16	24	11	12	40	17	14	14,17	34	12	11	22	37,38	11
ST161	Tibet_Shigatse_Tibetan	19	12	19	29	22	10	18	15	12	19	10	16	23	10	12	39	16	14	12,18	33	12	11	20	36,40	11
ST162	Tibet_Shigatse_Tibetan	17	12	20	27	19	9	17	14	12	19	10	17	24	11	14	35	18	15	13,16	31	12	12	22	34,37	11
ST163	Tibet_Shigatse_Tibetan	17	13	21	29	20	11	16	16	11	19	10	16	25	11	7	40	19	14	11,11	33	13	12	25	37,37	12
ST164	Tibet_Shigatse_Tibetan	18	12	20	29	21	11	22	15	11	19	10	15	27	10	10	35	19	14	17,17	30	12	11	30	36,36	11
ST165	Tibet_Shigatse_Tibetan	17	13	24	30	20	11	17	15	10	18	11	15	23	10	13	40	16	14	12,20	30	14	11	23	36,40	10
ST166	Tibet_Shigatse_Tibetan	17	13	24	30	20	11	17	15	10	18	11	15	23	10	13	40	16	14	12,20	30	14	11	23	36,40	10
ST167	Tibet_Shigatse_Tibetan	18	12	19	29	21	10	15	15	13	18	10	16	23	10	14	38	18	14	13,13	31	13	11	25	38,39	11
ST168	Tibet_Shigatse_Tibetan	17	13	21	29	22	9	16	15	11	19	10	15	25	11	7	36	20	14	11,14	30	12	12	28	36,37	12
ST169	Tibet_Shigatse_Tibetan	17	13	22	29	21	11	16	15	11	19	11	16	25	11	7	38	19	14	11,11	32	13	12	25	37,37	12
ST170	Tibet_Shigatse_Tibetan	16	12	20	28	22	10	16	15	12	18	10	17	23	10	14	35	19	14	13,13	31	13	11	24	35,38	11
ST171	Tibet_Shigatse_Tibetan	18	14	22	31	20	11	17	14	11	19	10	16	24	11	7	38	18	14	11,11	33	13	12	25	38,38	12
ST172	Tibet_Shigatse_Tibetan	18	14	22	31	20	11	17	14	11	19	10	16	24	11	7	38	18	14	11,11	33	13	12	25	38,39	12
ST173	Tibet_Shigatse_Tibetan	16	12	20	27	19	9	17	14	12	19	10	15	24	11	14	35	18	15	13,16	31	12	12	22	34,37	11
ST174	Tibet_Shigatse_Tibetan	18	12	21	26	18	10	18	14	12	20	10	15	23	11	14	34	18	15	13,19	33	12	12	23	36,37	11
ST175	Tibet_Shigatse_Tibetan	18	12	20	29	23	11	21	15	11	19	10	15	27	10	10	35	19	14	17,17	30	12	12	28	36,36	12
ST176	Tibet_Shigatse_Tibetan	19	12	21	28	20	9	20	15	12	20	10	15	23	11	14	36	18	16	13,17	35	12	13	23	35,37	11
ST177	Tibet_Shigatse_Tibetan	18	12	20	29	23	11	21	15	11	19	10	15	27	10	10	35	19	14	17,17	30	12	11	28	36,36	12
ST178	Tibet_Shigatse_Tibetan	16	13	20	29	21	11	16	15	11	19	10	16	25	11	7	38	21	14	11,14	30	13	11	28	36,36	12
ST179	Tibet_Shigatse_Tibetan	18	12	20	28	18	9	19	15	11	20	10	18	23	11	14	36	18	15	13,20	32	12	13	23	36,37	12
ST180	Tibet_Shigatse_Tibetan	19	13	21	29	22	10	19	15	11	17	10	15	24	10	11	41	21	14	14,18	29	13	10	29	36,36	11
ST181	Tibet_Shigatse_Tibetan	18	14	22	30	21	11	15	14	12	19	11	17	24	11	14	37	20	14	11,12	29	13	12	19	37,37	12
ST182	Tibet_Shigatse_Tibetan	17	14	24	30	20	11	15	14	12	19	11	15	24	11	14	37	18	14	11,12	28	13	12	22	37,37	12
ST183	Tibet_Shigatse_Tibetan	17	12	20	27	19	10	17	14	12	19	10	16	24	11	14	37	18	15	13,16	32	12	12	23	34,38	11
ST184	Tibet_Shigatse_Tibetan	20	13	21	29	20	10	16	15	11	17	10	15	23	10	11	40	17	14	13,18	29	12	10	26	36,36	12
ST185	Tibet_Shigatse_Tibetan	19	12	20	28	22	10	16	15	12	19	10	15	23	10	12	35	16	15	12,15	32	12	12	22	36,39	11
ST186	Tibet_Shigatse_Tibetan	18	13	21	29	19	11	15	15	11	19	10	16	25	11	7	38	19	14	11,11	32	13	13	25	36,39	11
ST187	Tibet_Shigatse_Tibetan	18	14	22	30	18	11	16	15	11	19	11	16	25	11	7	41	22	14	11,11	32	12	12	26	38,38	12
ST188	Tibet_Shigatse_Tibetan	17	13	21	29	22	10	18	14	11	17	10	17	24	10	11	39	16	14	14,19	28	13	10	27	36,36	12
ST189	Tibet_Shigatse_Tibetan	19	14	22	29	20	11	17	14	12	19	11	15	23	10	14	37	18	14	13,13	27	13	12	22	38,38	12

ST190 Tibet_Shigatse_Tibetan	18	12	21	29	22	11	20	15	11	19	10	16	25	10	10	36	19	14	16,17	31	13	11	28	36,37	12
ST191 Tibet_Shigatse_Tibetan	21	13	21	29	19	10	16	14	11	17	11	15	24	10	11	38	19	14	14,19	30	12	11	27	36,36	12
ST192 Tibet_Shigatse_Tibetan	19	13	22	29	19	11	17	15	11	19	10	15	25	11	7	38	20	14	11,11	33	13	13	27	38,39	12
ST193 Tibet_Shigatse_Tibetan	16	14	21	30	21	11	17	15	11	20	10	16	25	11	7	42	20	14	11,14	30	13	11	29	36,37	12
ST194 Tibet_Shigatse_Tibetan	19	13	22	29	22	11	16	15	11	19	10	16	25	11	7	42	20	14	11,15	30	13	12	28	36,38	12
ST195 Tibet_Shigatse_Tibetan	17	13	23	30	20	11	16	16	13	20	11	17	24	11	11	40	20	14	11,14	31	13	10	23	38,40	12
ST196 Tibet_Shigatse_Tibetan	18	14	25	30	15	11	15	14	12	19	11	15	24	11	13	36	18	14	11,12	28	14	12	22	37,37	12
ST197 Tibet_Shigatse_Tibetan	16	13	20	30	20	11	16	15	11	19	10	13	24	11	7	39	19	14	11,15	32	13	12	20	37,37	12
ST198 Tibet_Shigatse_Tibetan	17	13	22	28	19	9	18	16	11	20	10	15	23	10	14	37	19	14	12,18	32	13	12	25	37,39	11
ST199 Tibet_Shigatse_Tibetan	20	13	21	29	18	10	16	15	11	17	10	15	24	10	11	39	18	14	14,18	31	12	10	26	38,38	12
ST200 Tibet_Shigatse_Tibetan	17	12	21	28	21	9	17	14	12	20	10	15	23	11	14	35	16	15	13,15	33	12	13	22	35,38	11
ST201 Tibet_Shigatse_Tibetan	17	12	21	28	19	9	18	14	12	20	10	15	23	11	14	33	19	16	13,19	32	12	12	23	35,40	11
ST202 Tibet_Shigatse_Tibetan	20	14	21	30	21	11	15	15	11	22	10	15	23	10	11	39	16	14	11,18	30	13	11	26	37,38	12
ST203 Tibet_Shigatse_Tibetan	17	12	20	28	18	9	18	14	12	19	10	15	24	11	14	35	17	15	16,19	32	12	11	24	35,39	11
ST204 Tibet_Shigatse_Tibetan	18	14	22	30	19	11	16	16	11	19	11	15	25	11	7	40	19	15	11,11	32	13	12	26	37,38	12
ST205 Tibet_Shigatse_Tibetan	19	13	21	29	20	10	16	15	11	17	11	16	24	10	11	41	17	14	14,19	30	12	10	26	36,36	12
ST206 Tibet_Shigatse_Tibetan	19	14	21	30	20	10	18	14	11	17	11	14	23	10	11	39	17	14	14,19	30	12	10	25	37,37	12
ST207 Tibet_Shigatse_Tibetan	18	12	20	29	23	12	21	15	11	19	10	15	27	10	10	35	19	14	17,17	30	12	11	28	36,36	12
ST208 Tibet_Shigatse_Tibetan	20	12	21	28	18	9	18	15	12	20	10	15	23	11	14	37	19	15	13,17	33	12	12	24	34,38	11
ST209 Tibet_Shigatse_Tibetan	17	14	22	30	20	11	16	15	11	19	10	17	25	11	7	39	19	14	11,11	32	13	11	25	37,37	12
ST210 Tibet_Shigatse_Tibetan	19	12	21	28	22	9	16	14	11	20	10	15	23	11	14	36	19	15	13,19	31	12	12	23	35,38	11
ST211 Tibet_Shigatse_Tibetan	18	13	20	28	21	9	18	14	12	20	10	15	23	11	14	35	17	15	13,19	32	12	12	23	35,38	11
ST212 Tibet_Shigatse_Tibetan	17	13	20	30	19	10	20	15	13	21	11	17	24	11	7	38	18	14	11,13	33	11	12	27	39,39	13
ST213 Tibet_Shigatse_Tibetan	18	12	20	28	19	9	18	14	11	20	10	15	23	12	14	35	20	15	13,17	33	12	12	23	35,37	11
ST214 Tibet_Shigatse_Tibetan	18	13	21	30	21	11	16	15	11	19	10	16	25	11	7	41	20	14	11,14	29	13	11	29	37,38	12
ST215 Tibet_Shigatse_Tibetan	18	14	21	30	19	10	15	16	11	19	10	15	25	11	7	37	19	14	11,11	31	13	11	26	36,38	11
ST216 Tibet_Shigatse_Tibetan	18	12	20	28	18	9	18	14	12	20	10	15	23	11	14	37	18	15	13,19	32	12	13	23	35,39	11
ST217 Tibet_Shigatse_Tibetan	19	12	18	28	20	10	18	15	11	19	10	16	23	10	12	38	16	14	13,16	34	12	12	21	37,39	11
ST218 Tibet_Shigatse_Tibetan	17	12	20	28	20	9	16	14	12	20	10	15	23	11	14	35	18	15	13,18	32	12	13	23	35,39	11
ST219 Tibet_Shigatse_Tibetan	18	12	20	27	18	10	17	14	12	19	10	15	23	11	14	35	17	15	13,20	32	12	11	23	35,37	11
ST220 Tibet_Shigatse_Tibetan	17	13	23	30	18	11	17	16	12	20	10	15	24	11	11	43	19	14	11,14	32	13	10	23	37,39	12
ST221 Tibet_Shigatse_Tibetan	19	14	21	30	19	11	17	15	11	19	10	15	25	11	7	38	17	14	11,11	32	13	11	27	38,39	13
ST222 Tibet_Shigatse_Tibetan	17	14	21	31	19	11	16	16	12	20	10	15	24	11	7	39	15	14	12,15	31	12	12	22	37,37	12

ST223	Tibet_Shigatse_Tibetan	18	12	20	27	18	10	18	14	12	20	10	15	23	11	14	36	18	15	14,21	33	12	13	23	37,37	11
ST224	Tibet_Shigatse_Tibetan	17	13	21	29	22	11	16	15	11	19	10	15	23	11	7	38	18	14	11,14	30	13	11	29	38,38	12
ST225	Tibet_Shigatse_Tibetan	19	12	21	28	18	9	15	14	11	20	11	15	23	11	14	36	17	15	13,19	31	12	12	25	37,38	11
ST226	Tibet_Shigatse_Tibetan	18	12	21	28	22	13	20	15	11	20	10	15	25	10	10	34	20	14	16,16	28	12	11	28	37,40	11
ST227	Tibet_Shigatse_Tibetan	17	13	21	30	24	9	15	14	12	21	10	17	24	10	11	41	21	14	16,16	34	12	11	22	34,38	11
ST228	Tibet_Shigatse_Tibetan	18	12	20	28	18	9	20	14	12	20	10	15	23	11	14	37	18	15	14,20	31	12	12	23	35,38	11
ST229	Tibet_Shigatse_Tibetan	17	13	20	29	20	10	19	14	12	20	10	15	23	11	14	36	16	15	13,19	32	12	12	23	35,39	11
ST230	Tibet_Shigatse_Tibetan	18	13	22	29	20	11	17	14	11	17	10	15	25	10	11	39	19	14	15,15	30	12	10	27	36,36	11

Supplementary Table S2. The allele and corresponding frequencies in 172 Chamdo Kham Tibetans

Allele	DYS19	DYS389I	DYS389II	DYS390	DYS391	DYS392	DYS393	DYS437	DYS438	DYS439	DYS448	DYS449	DYS456	DYS458	DYS460	DYS481	DYS518	DYS533	DYS570	DYS576	DYS627	DYS635	YGATAH4	Allele	DYF387S1	Allele	DYS385
7						0.4826																		34,36	0.0058	10,11	0.0058
9					0.0233				0.0058						0.0872									34,39	0.0058	10,12	0.0116
10					0.7500	0.0698			0.1628	0.0291					0.3081			0.0058					0.0581	35,35	0.0116	10,13	0.0058
11		0.0058			0.2267	0.0233	0.0116		0.8256	0.3430					0.5174			0.3663					0.5291	35,36	0.0174	11,11	0.2326
12		0.2965				0.0233	0.3314		0.0058	0.5174					0.0814			0.5988		0.0058			0.3605	35,37	0.0523	11,12	0.1047
13	0.0174	0.2151				0.0291	0.6279	0.0058		0.1105					0.0058			0.0291					0.0465	35,38	0.0291	11,13	0.0174
14	0.3023	0.4360				0.2384	0.0291	0.7442					0.0988	0.0058									0.0058	35,39	0.0058	11,14	0.1802
15	0.5814	0.0465				0.1279		0.1570					0.4244	0.1105										36,36	0.0465	11,15	0.0233
16	0.0872					0.0058		0.0930					0.4360	0.2907					0.0407	0.1919	0.0058			36,37	0.2035	12,12	0.0349
17	0.0116										0.0233		0.0349	0.4070					0.0814	0.2442	0.0291			36,38	0.0756	12,13	0.0116
18											0.0872		0.0058	0.0814					0.2500	0.3547	0.1163			36,39	0.0058	12,15	0.0058
19											0.5814			0.0640		0.0174			0.3953	0.1512	0.2674	0.0233		36,41	0.0058	12,16	0.0058
20											0.2907			0.0349		0.0058			0.1802	0.0349	0.1919	0.2616		37,37	0.1919	12,17	0.0174
21											0.0116			0.0058					0.0349	0.0116	0.2093	0.4360		37,38	0.2151	12,19	0.0058
22				0.0291							0.0058					0.0814			0.0174	0.0058	0.1395	0.1744		37,39	0.0407	12,20	0.0116
23				0.3721												0.2151					0.0349	0.0698		38,38	0.0523	13,13	0.0116
24				0.1279												0.1047					0.0058	0.0349		38,39	0.0116	13,15	0.0116
25				0.4070												0.2093								39,39	0.0233	13,17	0.0058

26			0.0291	0.0581												0.0640										13,18	0.0174
27			0.1395	0.0058								0.0233				0.0756										13,19	0.0349
28			0.1163									0.0407				0.1453										13,20	0.1047
29			0.1860									0.1395				0.0465										13,21	0.0291
30			0.4128									0.2209				0.0291										14,14	0.0058
31			0.0872									0.2267														14,18	0.0058
32			0.0291									0.1802				0.0058										14,19	0.0174
33												0.0930					0.0116									14,20	0.0058
34												0.0698					0.0174									15,16	0.0233
35												0.0058					0.1163									16,16	0.0174
36																	0.1628									16,17	0.0174
37																	0.2733									17,17	0.0116
38																	0.2151									17,19	0.0058
39																	0.1337										
40																	0.0233										
41																	0.0233										
42																	0.0233										
Nall	5	5	7	6	3	8	4	4	4	4	6	9	5	8	5	12	10	4	7	8	9	6	5	-	18	-	31
GD	0.5658235	0.6774106	0.7571059	0.6792466	0.3878009	0.6911465	0.497824	0.4152727	0.2935537	0.6050592	0.5724874	0.8369373	0.622331	0.7299062	0.626683	0.8630491	0.8238814	0.5093159	0.7432341	0.7579219	0.8174895	0.7084863	0.5879913	-	0.8633891	-	0.8893649

Supplementary Table S3. The allele and corresponding frequencies in 230 Shigatse Ü-Tsang Tibetans

Allele	DYS19	DYS389I	DYS389II	DYS390	DYS391	DYS392	DYS393	DYS437	DYS438	DYS439	DYS448	DYS449	DYS456	DYS458	DYS460	DYS481	DYS518	DYS533	DYS570	DYS576	DYS627	DYS635	YGATAH4	Allele	DYF387S1	Allele	DYS385
7						0.2870																		34,36	0.0043	11,11	0.1826
8					0.0043										0.0043									34,37	0.0217	11,12	0.0522
9					0.0217				0.0087						0.2826			0.0043						34,38	0.0174	11,13	0.0391
10					0.7826	0.0652			0.3130	0.1087					0.2391			0.0217					0.0696	34,40	0.0130	11,14	0.0522
11					0.1913	0.1174	0.0087		0.6435	0.2696					0.4391			0.4913					0.4609	35,35	0.0043	11,15	0.0217
12		0.4348				0.0391	0.5000		0.0348	0.4522					0.0304			0.4565					0.4130	35,36	0.0217	11,17	0.0043
13	0.0348	0.2696				0.0652	0.4000			0.1478			0.0130	0.0043	0.0043			0.0261					0.0522	35,37	0.0565	11,18	0.0087
14	0.3609	0.2870				0.4000	0.0870	0.6609		0.0217			0.1000										0.0043	35,38	0.0826	11,21	0.0043
15	0.4870	0.0087				0.0261	0.0043	0.2913					0.5783	0.1304					0.0087	0.0043	0.0087			35,39	0.0435	12,12	0.0217
16	0.0870							0.0478			0.0043		0.2348	0.2565					0.0783	0.1043				35,40	0.0130	12,13	0.0130
17	0.0304			0.0043							0.0652		0.0652	0.2826					0.1217	0.2217	0.0087			35,41	0.0087	12,14	0.0043
18											0.0783		0.0087	0.1870					0.2913	0.3783	0.1696	0.0087		36,36	0.1043	12,15	0.0217
19				0.0043							0.4783			0.0391		0.0087			0.2609	0.2348	0.2478	0.0348		36,37	0.0652	12,16	0.0174
19.2																					0.0043			36,38	0.0261	12,17	0.0043
20											0.3000			0.0565		0.0174			0.1957	0.0478	0.2000	0.2826		36,39	0.0217	12,18	0.0130
21											0.0696			0.0304		0.0217			0.0391	0.0087	0.1217	0.4000		36,40	0.0174	12,19	0.0174
22				0.0391							0.0043			0.0130		0.1130			0.0043		0.1348	0.1826		36,43	0.0043	12,20	0.0087
23				0.3913												0.2739					0.0609	0.0435		37,37	0.1391	12,21	0.0043
24				0.2957												0.1087					0.0348	0.0435		37,38	0.1304	13,13	0.0217
25			0.0043	0.2043												0.1957					0.0087	0.0043		37,39	0.0348	13,14	0.0043
26			0.0087	0.0174												0.1000								37,40	0.0043	13,15	0.0087
27			0.0652	0.0435								0.0217				0.0522								38,38	0.0652	13,16	0.0261
28			0.2652									0.0522				0.0696								38,39	0.0478	13,17	0.0435
29			0.2696									0.0913				0.0261								38,40	0.0217	13,18	0.0478
30			0.3000									0.1652				0.0087								38,41	0.0043	13,19	0.0783
31			0.0783									0.2304				0.0043								39,39	0.0174	13,20	0.0217
32			0.0087									0.2348												39,40	0.0043	13,21	0.0087
33												0.1261					0.0043							40,41	0.0043	13,23	0.0043

34												0.0565					0.0304									14,14	0.0043
35												0.0130					0.1609									14,17	0.0043
36												0.0087					0.1870									14,18	0.0565
37																	0.1783									14,19	0.0478
37.2																	0.0174									14,20	0.0130
38																	0.1739									14,21	0.0043
39																	0.0826									15,15	0.0043
40																	0.0826									15,16	0.0043
41																	0.0565									15,19	0.0043
42																	0.0130									15,20	0.0043
43																	0.0130									15,21	0.0087
																										16,16	0.0174
																										16,17	0.0130
																										16,18	0.0043
																										16,19	0.0043
																										16,22	0.0043
																										17,17	0.0304
																										17,18	0.0087
																										17,22	0.0043
Nall	5	4	8	8	4	7	5	3	4	5	7	10	6	9	6	13	12	5	8	7	11	8	5	-	28	-	47
GD	0.6257	0.6587	0.7597	0.7171	0.3520	0.7364	0.5849	0.4782	0.4888	0.6917	0.6689	0.8373	0.5986	0.8000	0.6721	0.8466	0.8625	0.5514	0.7897	0.7426	0.8353	0.7249	0.6121	-	0.9288	-	0.9416

Supplementary Table S4. The demographical information of included populations

Population	Country	Linguistic family	N	Reference	Accession numbers
Shigatse-Ü-Tsang-Tibetans-(SUT)	China	Sino-Tibetan	230	This study	YA004562
Chamdo-Kham-Tibetans-(CKT)	China	Sino-Tibetan	172	This study	YA004561
Basque	Spain	Basque	207	1	YA003184
Chaoshan-Han	China	Sinitic	778	2	YA004148
Hulunbuir-Daur	China	Mongolic	203	3	YA004277
Hulunbuir-Mongolian	China	Mongolic	282	3	YA004555
Dezhou-Han	China	Sinitic	2000	4	YA004371
Eritrea-Cunama	Eritrea	Afroasiatic	19	5	YA004198-YA004207
Eritrea-Nara	Eritrea	Afroasiatic	15	5	YA004198-YA004207
Eritrea-Saho	Eritrea	Afroasiatic	94	5	YA004198-YA004207
Eritrea-Tigray	Eritrea	Afroasiatic	28	5	YA004198-YA004207
Eritrea-Tigre	Eritrea	Afroasiatic	5	5	YA004198-YA004207
Ethiopia-Amhara	Ethiopia	Afroasiatic	34	5	YA004198-YA004207
Ethiopia-Ethiopian-Jews	Ethiopia	Afroasiatic	22	5	YA004198-YA004207
Ethiopia-Oromo	Ethiopia	Afroasiatic	28	5	YA004198-YA004207
Ethiopia-Other-Ethiopians	Ethiopia	Afroasiatic	9	5	YA004198-YA004207
Ethiopia-Wolayta	Ethiopia	Afroasiatic	11	5	YA004198-YA004207
Ethiopia-Somali-Ethiopia	Ethiopia	Afroasiatic	10	5	YA004198-YA004207
Ethiopia-Tigray-Ethiopia	Ethiopia	Afroasiatic	5	5	YA004198-YA004207
Djibouti-Afar	Djibouti	Afroasiatic	20	5	YA004198-YA004207
Djibouti-Somali-Djibouti	Djibouti	Afroasiatic	34	5	YA004198-YA004207
Kenya-Borana	Kenya	Afroasiatic	7	5	YA004198-YA004207
Kenya-Luhya	Kenya	Afroasiatic	51	5	YA004198-YA004207
Kenya-Maasai	Kenya	Afroasiatic	45	5	YA004198-YA004207
Kenya-Other-Bantu	Kenya	Afroasiatic	11	5	YA004198-YA004207
Kenya-Other-Nilo-Saharans	Kenya	Afroasiatic	9	5	YA004198-YA004207
Kenya-Somali-Kenya	Kenya	Afroasiatic	5	5	YA004198-YA004207
Espírito-Santo	Brazil	Afroasiatic	409	6	YA004541
Gansu-Dongxiang	China	Mongolic	526	7	YA004178
Guangdong-Han	China	Sinitic	1007	8	YA004066
Guangxi-Zhuang	China	Tai-Kadai	2314	9	YA004208

Guizhou-Gelao	China	Tai-Kadai	297	10	YA004331
Guizhou-Miao	China	Hmong–Mien	220	10	YA004332
Hainan-Li	China	Tai-Kadai	102	11	YA004364
Kazakh	Kazakhstan	Kipchak Turkic	300	12	YA004316 and YA004322
Henan-Han	China	Sinitic	1413	13	YA004150
HongKong-Chinese	China	Sinitic	402	14	YA004375
Gansu-Hui	China	Sinitic	377	15	YA004305
Hubei-Tujia	China	Sino-Tibetan	391	15	YA004306
Nantong-Han	China	Sinitic	1394	16	YA004376
Moroccan-Jews	Morocco	Afroasiatic	31	17	YA004351-YA004356
Morocco-Asni-Berbers	Morocco	Afroasiatic	53	17	YA004351-YA004356
Morocco-Bouhria-Berbers	Morocco	Afroasiatic	66	17	YA004351-YA004356
Morocco-Souss-Berbers	Morocco	Afroasiatic	28	17	YA004351-YA004356
Morocco-Ouarzazate-Berbers	Morocco	Afroasiatic	28	17	YA004351-YA004356
Algeria-Mozabite-Berbers	Algeria	Afroasiatic	64	17	YA004351-YA004356
Libya-Libyan-Arabs	Libya	Afroasiatic	63	17	YA004351-YA004356
Libya-Libyan-Jews	Libya	Afroasiatic	24	17	YA004351-YA004356
Egypt-Northern-Egyptians	Egypt	Afroasiatic	35	17	YA004351-YA004356
Egypt-Siwa-Egyptians-Berbers	Egypt	Afroasiatic	54	17	YA004351-YA004356
Egypt-Baharia-Egyptians	Egypt	Afroasiatic	31	17	YA004351-YA004356
Poland	Poland	Indo-European	586	18	YA004147
Qinghai-Tibetan	China	Sino-Tibetan	511	19	YA004181
Centra-Saudi-Arabia	Saudi Arabia	Arabic	107	20	YA004270
East-Saudi-Arabia	Saudi Arabia	Arabic	93	20	YA004271
North-Saudi-Arabia	Saudi Arabia	Arabic	96	20	YA004272
South-Saudi-Arabia	Saudi Arabia	Arabic	179	20	YA004273
West-Saudi-Arabia	Saudi Arabia	Arabic	28	20	YA004274
Upper-Austrian	Austrian	Indo-European	225	21	YP000295
Salzburg-Austrian	Austrian	Indo-European	200	21	YP000352
Guizhou-Yi	China	Sino-Tibetan	217	22	YA004512
Sichuan-Yi	China	Sino-Tibetan	227	22	YA004513
Yunnan-Yi	China	Sino-Tibetan	66	22	YA004513

Refences

1 **Garcia, O. et al. Data for 27 Y-chromosome STR loci in the Basque Country autochthonous population. Forensic Sci Int Genet 20, e10-e12, doi:10.1016/j.fsigen.2015.09.010 (2016).**

2 Zhang, J. et al. Haplotype data for 27 Y-chromosomal STR loci in the Chaoshan Han population, South China. Forensic Sci Int Genet 31, e54-e56, doi:10.1016/j.fsigen.2017.08.003 (2017).

3 Wang, C. Z. et al. Genetic polymorphisms of 27 Yfiler((R)) Plus loci in the Daur and Mongolian ethnic minorities from Hulunbuir of Inner Mongolia Autonomous Region, China. Forensic Sci Int Genet 40, e252-e255, doi:10.1016/j.fsigen.2019.02.003 (2019).

4 Zhang, J. et al. Genetic polymorphisms of 27 Y-STR loci in the Dezhou Han population from Shandong province, Eastern China. Forensic Sci Int Genet 39, e26-e28, doi:10.1016/j.fsigen.2018.11.021 (2019).

5 Iacovacci, G. et al. Forensic data and microvariant sequence characterization of 27 Y-STR loci analyzed in four Eastern African countries. Forensic Sci Int Genet 27, 123-131, doi:10.1016/j.fsigen.2016.12.015 (2017).

6 Santos Stange, V. et al. Stratification among European descent and admixed Brazilian populations of Espirito Santo for 27 Y-STRs. Forensic Sci Int Genet, doi:10.1016/j.fsigen.2019.03.019 (2019).

7 Wang, J. et al. Haplotype structure of 27 Yfiler((R))Plus loci in Chinese Dongxiang ethnic group and its genetic relationships with other populations. Forensic Sci Int Genet 33, e13-e16, doi:10.1016/j.fsigen.2017.12.014 (2018).

8 Wang, Y. et al. Genetic polymorphisms and mutation rates of 27 Y-chromosomal STRs in a Han population from Guangdong Province, Southern China. Forensic Sci Int Genet 21, 5-9, doi:10.1016/j.fsigen.2015.09.013 (2016).

9 Guo, F., Li, J., Chen, K., Tang, R. & Zhou, L. Population genetic data for 27 Y-STR loci in the Zhuang ethnic minority from Guangxi Zhuang Autonomous Region in the south of China. Forensic Sci Int Genet 27, 182-183, doi:10.1016/j.fsigen.2016.11.009 (2017).

10 Liu, Y. et al. Haplotypes of 27 Y-STRs analyzed in Gelao and Miao ethnic minorities from Guizhou Province, Southwest China. Forensic Sci Int Genet 40, e264-e267, doi:10.1016/j.fsigen.2019.03.002 (2019).

11 Song, M. et al. Forensic characteristics and phylogenetic analysis of both Y-STR and Y-SNP in the Li and Han ethnic groups from Hainan Island of China. Forensic Sci Int Genet 39, e14-e20, doi:10.1016/j.fsigen.2018.11.016 (2019).

12 Zhabagin, M. et al. Development of the Kazakhstan Y-chromosome haplotype reference database: analysis of 27 Y-STR in Kazakh population. Int J Legal Med, doi:10.1007/s00414-018-1859-8 (2018).

13 Wang, L. et al. Genetic population data of Yfiler Plus kit from 1434 unrelated Hans in Henan Province (Central China). Forensic Sci Int Genet 22, e25-e27, doi:10.1016/j.fsigen.2016.02.009 (2016).

14 Ip, S. C. Y., Lin, S. W. & Lam, T. T. Haplotype data of 27 Y-STR loci in Hong Kong Chinese. Forensic Sci Int Genet 38, e14-e15, doi:10.1016/j.fsigen.2018.11.001 (2019).

15 Liu, Y. et al. Haplotype data of 27 Y-STRs analyzed in the Hui and Tujia ethnic minorities from China. Forensic Sci Int Genet 35, e7-e9, doi:10.1016/j.fsigen.2018.04.006 (2018).

16 Tao, R. et al. Genetic characterization of 27 Y-STR loci analyzed in the Nantong Han population residing along the Yangtze Basin. Forensic Sci Int Genet 39, e10-e13, doi:10.1016/j.fsigen.2018.11.015 (2019).

17 D'Atanasio, E. et al. Rapidly mutating Y-STRs in rapidly expanding populations: Discrimination power of the Yfiler Plus multiplex in northern Africa. Forensic Sci Int Genet 38, 185-194, doi:10.1016/j.fsigen.2018.11.002 (2019).

18 Spolnicka, M. et al. Intra- and inter-population analysis of haplotype diversity in Yfiler((R)) Plus system using a wide set of representative data from Polish population. Forensic

Sci Int Genet 28, e22-e25, doi:10.1016/j.fsigen.2017.01.014 (2017).
19 Cao, S. et al. Genetic portrait of 27 Y-STR loci in the Tibetan ethnic population of the Qinghai province of China. Forensic Sci Int Genet 34, e18-e19, doi:10.1016/j.fsigen.2018.02.005 (2018).
20 Khubrani, Y. M., Wetton, J. H. & Jobling, M. A. Extensive geographical and social structure in the paternal lineages of Saudi Arabia revealed by analysis of 27 Y-STRs. Forensic Sci Int Genet 33, 98-105, doi:10.1016/j.fsigen.2017.11.015 (2018).
21 Pickrahn, I. et al. Yfiler((R)) Plus amplification kit validation and calculation of forensic parameters for two Austrian populations. Forensic Sci Int Genet 21, 90-94, doi:10.1016/j.fsigen.2015.12.014 (2016).
22 Fan, G. Y. et al. Forensic and phylogenetic analyses among three Yi populations in Southwest China with 27 Y chromosomal STR loci. Int J Legal Med, doi:10.1007/s00414-018-1984-4 (2018).

Supplementary Table S5. Genetic distances (Rst) and corresponding p values between Ü-Tsang Tibetans and Kham Tibetans and 19 Chinese reference populations

Population	Tibet-Chamdo-Tibetan	Tibet-Shigatse-Tibetan	Inner Mongolia-China-Daur	Gansu-China-Dongxiang	Guizhou-China-Gelao	Beijing-China-Han	Chaoshan-China-Han	China-Han	Chongqing-China-Han	Hainan-China-Han	Jining-China-Han	Shanghai-China-Han	Shenzhen-China-Han	Gansu-China-Hui	Yanbian-China-Korean	Hainan-China-Li	Hainan-China-Lingao	Guizhou-China-Miao	Gansu-China-Tibetan	Qinghai-China-Tibetan	Hubei-China-Tujia
Tibet-Chamdo-Tibetan	-	0.0001	0.0000	0.0000	0.0000	0.0000	0.0000	0.0000	0.0000	0.0000	0.0000	0.0000	0.0000	0.0000	0.0000	0.0000	0.0000	0.0000	0.0000	0.0000	0.0000
Tibet-Shigatse-Tibetan	0.0291	-	0.0000	0.0000	0.0000	0.0000	0.0000	0.0000	0.0000	0.0000	0.0000	0.0000	0.0000	0.0000	0.0000	0.0000	0.0000	0.0000	0.0183	0.0000	0.0000
Inner Mongoli -Daur	0.1575	0.1234	-	0.0000	0.0000	0.0000	0.0000	0.0000	0.0000	0.0000	0.0000	0.0000	0.0000	0.0000	0.0000	0.0000	0.0000	0.0000	0.0000	0.0000	0.0000
Gansu -Dongxiang	0.1002	0.0584	0.0551	-	0.0000	0.0000	0.0000	0.0000	0.0000	0.0000	0.0000	0.0000	0.0000	0.0019	0.0000	0.0000	0.0000	0.0000	0.0000	0.0000	0.0000
Guizhou-China-Gelao	0.1392	0.0635	0.0702	0.0563	-	0.0000	0.0000	0.0000	0.0080	0.0000	0.0000	0.0000	0.0001	0.0000	0.0000	0.0000	0.0000	0.0012	0.0000	0.0000	0.0000
Beijing-China-Han	0.1478	0.0548	0.1084	0.0654	0.0148	-	0.0002	0.0103	0.0016	0.0000	0.3649	0.0000	0.0095	0.0000	0.0000	0.0000	0.0000	0.0508	0.0000	0.0000	0.0000
Chaoshan-China-Han	0.1680	0.0722	0.1356	0.0863	0.0189	0.0072	-	0.0000	0.0006	0.0000	0.0000	0.0000	0.0256	0.0000	0.0000	0.0000	0.0000	0.0576	0.0000	0.0000	0.0000
China-Han	0.1567	0.0632	0.1145	0.0754	0.0137	0.0034	0.0055	-	0.0012	0.0000	0.0000	0.0000	0.0005	0.0000	0.0000	0.0000	0.0000	0.0043	0.0000	0.0000	0.0000
Chongqing-China-Han	0.1524	0.0626	0.0989	0.0655	0.0045	0.0052	0.0039	0.0043	-	0.0010	0.0000	0.0000	0.0638	0.0000	0.0000	0.0000	0.0000	0.4416	0.0000	0.0000	0.0000
Hainan-China-Han	0.1740	0.0807	0.1070	0.0759	0.0116	0.0105	0.0078	0.0143	0.0039	-	0.0000	0.0000	0.5627	0.0000	0.0000	0.0000	0.0002	0.0238	0.0000	0.0000	0.0000
Jining-China-Han	0.1522	0.0572	0.1115	0.0622	0.0211	0.0001	0.0110	0.0075	0.0093	0.0138	-	0.0000	0.0025	0.0000	0.0000	0.0000	0.0000	0.0068	0.0000	0.0000	0.0000
Shanghai-China-Han	0.1684	0.0738	0.1245	0.0831	0.0192	0.0106	0.0122	0.0076	0.0097	0.0163	0.0154	-	0.0000	0.0000	0.0000	0.0000	0.0000	0.0000	0.0000	0.0000	0.0000

Shenzhen-China-Han	0.1708	0.0731	0.1173	0.0784	0.0154	0.0073	0.0051	0.0118	0.0036	-0.0007	0.0084	0.0139	-	0.0000	0.0000	0.0000	0.0081	0.2610	0.0000	0.0000	0.0000
Gansu-China-Hui	0.0924	0.0369	0.0659	0.0053	0.0320	0.0349	0.0534	0.0436	0.0371	0.0480	0.0328	0.0506	0.0471	-	0.0000	0.0000	0.0000	0.0000	0.0000	0.0000	0.0000
Yanbian-China-Korean	0.2174	0.1206	0.1606	0.1119	0.0604	0.0461	0.0508	0.0595	0.0502	0.0497	0.0425	0.0627	0.0398	0.0832	-	0.0000	0.0000	0.0000	0.0000	0.0000	0.0000
Hainan-China-Li	0.2174	0.1364	0.0926	0.0921	0.0296	0.0744	0.0671	0.0768	0.0448	0.0364	0.0767	0.0722	0.0493	0.0750	0.1105	-	0.0000	0.0000	0.0000	0.0000	0.0000
Hainan-China-Lingao	0.1879	0.0997	0.1010	0.0757	0.0119	0.0269	0.0197	0.0287	0.0120	0.0055	0.0312	0.0297	0.0089	0.0522	0.0606	0.0175	-	0.0000	0.0000	0.0000	0.0000
Guizhou-China-Miao	0.1573	0.0627	0.1107	0.0667	0.0117	0.0032	0.0027	0.0066	-0.0001	0.0038	0.0047	0.0118	0.0013	0.0362	0.0430	0.0554	0.0160	-	0.0000	0.0000	0.0000
Gansu-China-Tibetan	0.0281	0.0060	0.1359	0.0702	0.0883	0.0769	0.0936	0.0881	0.0861	0.0993	0.0782	0.1003	0.0923	0.0542	0.1347	0.1517	0.1189	0.0849	-	0.0000	0.0000
Qinghai-China-Tibetan	0.1082	0.0989	0.1289	0.0866	0.1566	0.1705	0.1994	0.1758	0.1745	0.1895	0.1625	0.1880	0.1929	0.0878	0.2222	0.2054	0.1938	0.1850	0.1120	-	0.0000
Hubei-China-Tujia	0.1107	0.0782	0.0721	0.0768	0.0397	0.0699	0.0818	0.0659	0.0618	0.0808	0.0835	0.0792	0.0910	0.0611	0.1395	0.0947	0.0775	0.0737	0.1006	0.1547	-

Supplementary Table S6. Genetic distances (Rst) and corresponding p values between two Tibetans and 17 Chinese reference populations residing in the Tibet harsh environment region and the surroundings of Himalayan.

Population	Chamdo_Tibetan	Shigatse_Tibetan	Afghanistan-Hazara	Xinjiang-Kazakh	Xinjiang-Uighur	Guizhou-Yi	Sichuan-Yi	Yunnan-Yi	Madhya-Pradesh-Indian	Assam-Kachari	Kerala-Keralite	Andhra-Pradesh-Thoti	Kazakhstan-Kazakh	Pakistan-Gujjar	Balochistan-Hazara	Pakistan-Kohistani	Tharklani-Pashtun	Uthmankheil-Pashtun	Yousafzai-Pashtun
Chamdo_Tibetan	-	0.0004	0.0000	0.0000	0.0000	0.0000	0.0000	0.0000	0.0000	0.0029	0.0839	0.0052	0.0000	0.0000	0.0000	0.0000	0.0000	0.0000	0.0001
Shigatse_Tibetan	0.0291	-	0.0000	0.0000	0.0000	0.0000	0.0000	0.0000	0.0000	0.0201	0.3169	0.0122	0.0000	0.0000	0.0000	0.0001	0.0000	0.0000	0.0000
Afghanistan-Hazara	0.1458	0.1196	-	0.0000	0.0000	0.0000	0.0000	0.0000	0.0000	0.0031	0.1104	0.0205	0.0018	0.0000	0.0000	0.0000	0.0000	0.0000	0.0000
Xinjiang-Kazakh	0.1580	0.0878	0.0627	-	0.0000	0.0000	0.0000	0.0000	0.0000	0.0541	0.3384	0.0305	0.0000	0.0001	0.0000	0.0001	0.0000	0.0000	0.0000
Xinjiang-Uighur	0.1121	0.0633	0.0491	0.0634	-	0.0000	0.0000	0.0000	0.0000	0.0400	0.7756	0.2616	0.0000	0.0000	0.0000	0.0085	0.0000	0.0000	0.0000
Guizhou-Yi	0.1592	0.0712	0.1049	0.0502	0.0556	-	0.0000	0.0004	0.0000	0.1801	0.4865	0.0046	0.0000	0.0000	0.0000	0.0000	0.0000	0.0000	0.0000
Sichuan-Yi	0.1545	0.0561	0.1572	0.0670	0.0886	0.0212	-	0.0000	0.0000	0.1416	0.3095	0.0002	0.0000	0.0000	0.0000	0.0000	0.0000	0.0000	0.0000
Yunnan-Yi	0.1799	0.1092	0.0997	0.0553	0.0675	0.0362	0.0937	-	0.0000	0.0537	0.4821	0.0289	0.0000	0.0000	0.0000	0.0004	0.0000	0.0000	0.0000
Madhya-Pradesh-Indian	0.1694	0.1146	0.0966	0.1055	0.0191	0.0940	0.1308	0.0991	-	0.0458	0.8104	0.5066	0.0000	0.0000	0.0000	0.0248	0.0000	0.0000	0.0000
Assam-Kachari	0.2147	0.1189	0.1584	0.0964	0.0692	0.0317	0.0390	0.0962	0.0746	-	0.6124	0.2507	0.0047	0.0806	0.0001	0.0037	0.0000	0.0001	0.0006
Kerala-Keralite	0.1248	0.0260	0.0938	0.0246	-0.0568	-0.0119	0.0229	-0.0095	-0.0706	-0.0450	-	0.7996	0.1493	0.3160	0.0104	0.6602	0.0295	0.0058	0.0504
Andhra-Pradesh-Thoti	0.1502	0.1138	0.0819	0.0999	0.0123	0.1140	0.1536	0.0984	-0.0071	0.0428	-0.0885	-	0.0061	0.0552	0.0007	0.0870	0.0012	0.0003	0.0067
Kazakhstan-Kazakh	0.1292	0.1058	0.0090	0.0488	0.0601	0.0972	0.1393	0.0914	0.1111	0.1343	0.0695	0.0975	-	0.0000	0.0000	0.0000	0.0000	0.0000	0.0000

Pakistan-Gujjar	0.2487	0.1817	0.1282	0.1381	0.1034	0.1155	0.1562	0.1595	0.1290	0.0986	0.0382	0.1075	0.1312	-	0.0000	0.0005	0.0000	0.0000	0.0000
Balochistan-Hazara	0.1137	0.1324	0.0936	0.1526	0.1330	0.1948	0.2177	0.2176	0.1807	0.2565	0.2394	0.1822	0.0661	0.2450	-	0.0000	0.0000	0.0000	0.0000
Pakistan-Kohistani	0.1876	0.1351	0.1436	0.1530	0.0393	0.1198	0.1730	0.1186	0.0311	0.1914	-0.0546	0.0643	0.1479	0.1940	0.2502	-	0.0002	0.0000	0.0010
Tharklani-Pashtun	0.1853	0.1946	0.2450	0.2840	0.1413	0.2776	0.3038	0.2911	0.1633	0.5412	0.3848	0.2950	0.2430	0.4512	0.3085	0.2140	-	0.0000	0.6729
Uthmankheil-Pashtun	0.4093	0.3548	0.2788	0.3191	0.2894	0.3120	0.3766	0.3123	0.3262	0.5457	0.5361	0.4611	0.2899	0.4288	0.4977	0.4921	0.7308	-	0.0000
Yousafzai-Pashtun	0.1558	0.1610	0.2079	0.2466	0.1079	0.2401	0.2650	0.2576	0.1347	0.4390	0.2581	0.2167	0.2073	0.3861	0.2692	0.1578	-0.0164	0.6763	-

Supplementary Note 1:

```
#map for 19 Himalayan and adjacent populations
mydata<-read.table("C:/Users/Guanglin He/Desktop/All/mydata.csv",header=TRUE,sep=",")
library("ggrepel")
library("ggplot2")
library(maps)
library(mapdata)
china <- map(database = "world",xlim = c(47,135), ylim = c(0,50),fill = TRUE, col = 8, panel.first = grid())
ggplot() +
  geom_text_repel(data = mydata, aes(x = long, y = lat, label = text, color = population,size=16)) + labs(x = 'Longitude', y = 'Latitude')+
  geom_path(data = china, aes(long, lat, group = group), color = 'black', show.legend = F, linemitre=1,size=0.01, alpha = 0.8) +
  geom_point(data = mydata, aes(x = long, y = lat, size=16, shape = Ethnicity,color = population)) +
  theme_bw() +
  theme(legend.position = "left",legend.background = element_rect(fill="lightblue",    size=0.5, linetype="solid", colour ="darkblue"))+
  scale_shape_manual(values = c(15,16,17,18,19,15,16,17,18,19,15,16,17,18,19,15,16,17,18,19))+
  scale_colour_gradientn(colours = c("green","red"),values=c(1.0,0.8,0.6,0.4,0.2,0))
```

Supplementary Note 2:

```
#map for genetic similarities and differences between two newly investigated Tibetan populations and 19 Chinese reference populations
mydata<-read.table("F:/已写文章/西藏藏族 Y27/3 Nationwide population comparisons/mydata.csv",header=TRUE,sep=",")
library("ggrepel")
library("ggplot2")
library(maps)
library(mapdata)
map("china", col = "blue", ylim = c(17, 57), panel.first = grid())
china <- map("china", col = "blue", ylim = c(18, 56), panel.first = grid())
map
ggplot() +
  geom_text_repel(data = mydata, aes(x = long, y = lat, label = text, color = Ethnicity,size=16)) + labs(x = 'Longitude', y = 'Latitude')+
  geom_path(data = china, aes(long, lat, group = group), color = 'black', show.legend = F, linemitre=1,size=0.01, alpha = 0.8) +
  geom_point(data = mydata, aes(x = long, y = lat, size=16, shape = Ethnicity,color = Ethnicity)) +
  theme_bw() +
  theme(legend.position = "left",legend.background = element_rect(fill="lightblue",    size=0.5, linetype="solid", colour ="darkblue"))+
  scale_shape_manual(values = c(15,16,17,18,19,15,16,17,18,19,15,16,17,18,19,15,16,17,18,19)) +
  scale_colour_gradientn(colours = c("green","red"),values=c(1.0,0.8,0.6,0.4,0.2,0))
```
